# Effects of Active‐Site Modification and Quaternary Structure on the Regioselectivity of Catechol‐*O*‐Methyltransferase

**DOI:** 10.1002/anie.201508287

**Published:** 2016-01-21

**Authors:** Brian J. C. Law, Matthew R. Bennett, Mark L. Thompson, Colin Levy, Sarah A. Shepherd, David Leys, Jason Micklefield

**Affiliations:** ^1^ School of Chemistry & Manchester Institute of BiotechnologyUniversity of Manchester131 Princess StreetManchesterM1 7DNUK

**Keywords:** biocatalysis, enzyme structure, methyltransferases, protein engineering, regioselectivity

## Abstract

Catechol‐*O*‐methyltransferase (COMT), an important therapeutic target in the treatment of Parkinson's disease, is also being developed for biocatalytic processes, including vanillin production, although lack of regioselectivity has precluded its more widespread application. By using structural and mechanistic information, regiocomplementary COMT variants were engineered that deliver either *meta*‐ or *para*‐methylated catechols. X‐ray crystallography further revealed how the active‐site residues and quaternary structure govern regioselectivity. Finally, analogues of AdoMet are accepted by the regiocomplementary COMT mutants and can be used to prepare alkylated catechols, including ethyl vanillin.

Rational design or directed evolution has often been used to improve or alter the enantioselectivity or regioselectivity of enzymes for biocatalytic applications.^1^ Nature has evolved regiocomplementary enzymes for certain transformations;^2^ however, where no suitable native enzyme exists, engineering regiocomplementary variants to deliver defined regioisomeric products is an enticing prospect. One example where the lack of enzyme regioselectivity limits potential commercial productivity is the engineered biosynthesis of the flavor and fragrance agent vanillin **2 a**.^3^ Catechol‐*O*‐methyltransferase (COMT) has been used to methylate 3,4‐dihydroxybenzaldehyde (DHBAL, **1 a**) and 3,4‐dihydroxybenzoic acid (DHBA, **1 b**) in engineered strains of *Escherichia coli* and fission yeast for the production of vanillin **2 a** from glucose (Figure 1 A and Figure S1 in the Supporting Information).^3^, ^4^ However, the lack of regioselectivity of COMT results in a mixture of **2 a** and the undesired *para*‐methylated isovanillin **3 a**, which are difficult to separate. Regioselective COMT variants would greatly improve the more sustainable biochemical route for the production of vanillin in a microbial host. Additionally, regiocomplementary variants that deliver either *meta*‐ or *para*‐methylated catechols would be of interest because such moieties are found within a number of pharmaceuticals (see Figure S2 for examples).

**Figure 1:**
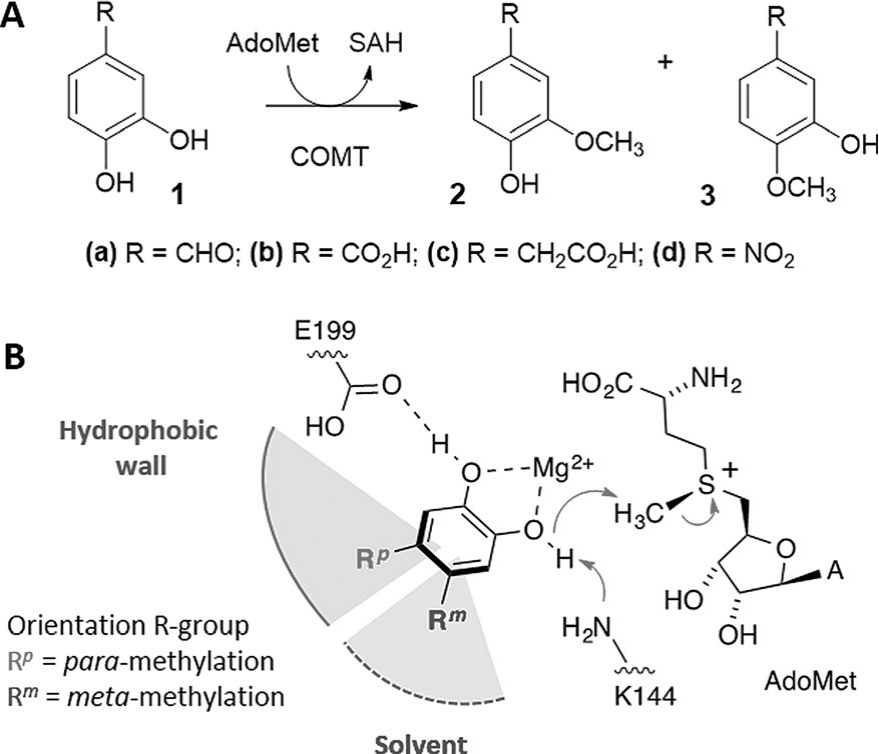
A) COMT methylation of substrates (**1**) to give *meta* (**2**) and *para* (**3**) products. B) mechanism of COMT‐catalyzed methylation of catechols using the cofactor *S*‐adenosyl‐l‐methionine (AdoMet). Coordination of Mg^2+^ by the catechol hydroxy groups lowers their p*K*_a_ values, thus aiding deprotonation of the hydroxy group closest to the AdoMet methyl group by K144. E199 appears to hydrogen bond with the other hydroxy group. The orientation of the R group in the active site determines whether the *meta*‐ or *para*‐hydroxy group is methylated.

Structural and mechanistic information suggest that the regioselectivity of COMT can be attributed to the concerted action of a “hydrophobic wall” found in the active site of the enzyme,^5^ and the substrate properties (Figure 1 B).^6^ Polar and ionisable substituents (R groups), such as those found on catecholamines, the natural substrates for COMT, are more likely to orientate out of the active site and into the solvent, thereby resulting in *meta*‐methylation.6a,6b For example, norepinephrine shows a *meta*/*para* ratio of 7:1.6b Conversely, *para*‐methylation is likely to be more evident for substrates with neutral or more hydrophobic substituents that can be orientated towards the hydrophobic wall.6a,6b The relative p*K*_a_ values and nucleophilicity of the two hydroxy groups could also affect the regiochemical outcome of the reaction.5a,5b, 6c

The COMT‐catalyzed methylation of catechols (**1**) is irreversible, resulting in a kinetically controlled ratio of regioisomers (**2** and **3**) that remains unchanged over time (Figure S3). Consequently, to explore and improve the regioselectivity of COMT, we carried out active‐site mutagenesis on residues most likely to affect substrate orientation: W38^7^ and Y200 of the hydrophobic pocket; V173, which is positioned directly above the catechol binding site; and E199 and K144, which may interact with the catechol hydroxy groups. The percentage conversions and regioselectivities of the COMT mutants were determined for substrates **1 a**–**d** (Figure 2, Table S1, and Figure S4). The W38 mutants showed an increase in *meta*‐methylation with **1 a** and 4‐nitrocatechol (NOC, **1 d**) with W38D exhibiting the highest regioselectivity: approximately +93 % regioisomeric excess (*re*), and around 50 % conversion, compared to *re* values of +54 % (**1 a**) and +72 % (**1 d**) with wild‐type (WT) COMT. Interestingly, with **1 b** and 3,4‐dihydroxyphenylacetic acid (DHPA, **1 c**), the W38 mutants (and Y200R) showed a shift toward increased *para*‐methylation. Notably, W38K and W38R mutants favored *para*‐ over *meta*‐methylation, with W38R exhibiting the highest *re* values in favor of *para* product: −39 % (**1 b**) and −40 % (**1 c**). The reversal in regioselectivity compared to WT [+58 % (**1 b**) and +82 % (**1 c**)], is likely due to the formation of a salt bridge between the basic sidechain of Arg/Lys and the carboxylate of **1 b** and **1 c**, which pulls the R group towards the hydrophobic pocket, thus leading to *para*‐methylation (Figure 1 B). The Y200 mutants generally showed increased *re* values in favor of the *meta* product with all substrates, with Y200L exhibiting the greatest *meta*‐selectivity with **1 a**–**d** whilst retaining relative activity similar to that of the WT with **1 a** and **1 d**. Significant shifts towards *para*‐selectivity were also observed for the K144A/V173Y mutant with **1 a** (−0.6 % *re*) and **1 d** (−40 % *re*). Kinetic parameters were determined for mutants that showed significant shifts in regioselectivity (Table S2), with mutations at the W38 position appearing to perturb substrate binding to a greater extent than those at the Y200 position (full details of kinetics are described in the Supporting Information). Interestingly, mutation of the proposed catalytic Lys residue (K144A) does not abolish COMT activity (Figure 2 and Table S2), thus suggesting that whilst this residue may participate in deprotonating the catechol hydroxy group, it is not essential. The effect of pH on COMT activity (Figure S5) shows that whilst the WT COMT activity is independent of pH, the K144A mutant activity increases significantly with increasing pH. Presumably, in the absence of a general base (K144), the solvent can deprotonate the hydroxy group, aided by coordination to Mg^2+^.

**Figure 2:**
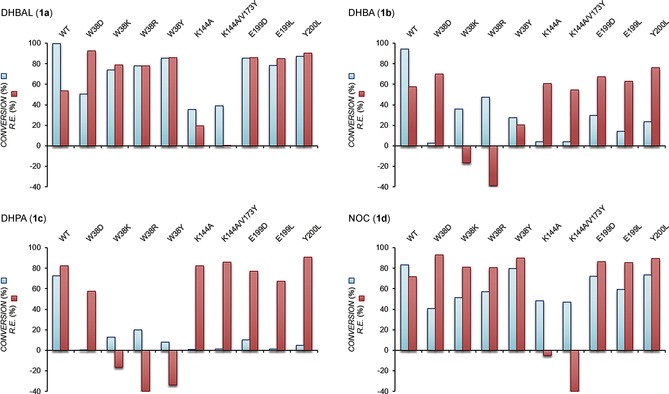
Activities and regioselectivities of WT COMT and selected mutants. The conversion (blue bar) was calculated as the percentage of initial substrate converted after 20 min to both regioisomeric products combined. The regioisomeric excess (*re*; red bar) was calculated as the percentage excess of the *meta* regioisomer over the *para* regioisomer. Positive *re* values denote *meta*‐selectivity, whereas negative *re* values denote *para*‐selectivity. For all other mutants, see Tables S1–S4.

The Y200L mutant, which shows both high *meta*‐regioselectivity and activity (Figure 2), was crystallized with bound *S*‐adenosyl methionine (AdoMet) and 3,5‐dinitrocatechol (DNC) for active‐site comparison with WT COMT with DNC (PDB ID: 1VID). The X‐ray crystal structure was solved to 1.6 Å resolution and is dimeric (PDB ID: 5FHR; Table S3). A preliminary structure of Y200L with *S*‐adenosyl homocysteine (SAH) and **1 a** was also obtained at 1.9 Å resolution (Figure S6). In these structures, the adjacent E199 residue has moved 9.6 Å out of the active site (Figure 3 A, C) compared to the previously published WT monomer structure (PDB ID: 1VID, Figure 3 B, C). This suggests that the lack of H‐bonding between E199 and the aldehyde group of a bound substrate **1 a** would result in preferential aldehyde‐group interaction with water molecules in the solvent, thereby leading to the high observed *re* in favor of the *meta* product (+90 %; Figure 3 D). Conversely, in the WT structure, Y200 is predicted to hydrogen bond with N41 (not shown), thereby aiding in maintenance of the hydrophobic wall. The E199 residue is thus orientated towards the catechol in the active site, and would be within hydrogen‐bonding distance of the aldehyde group of **1 a** if it were bound instead (Figure 3 E). Interaction between E199 and the aldehyde group of **1 a** could thus stabilize the substrate binding mode required for *para*‐methylation, which would account for the lower *re* observed (+58 %) with WT COMT. To further investigate the role of E199, this amino acid was replaced with a Leu residue of similar size but unable to interact with the aldehyde group of **1 a**. This resulted in an *re* in favor of the *meta* product (+85 %; Figure 2), which is consistent with the substrate aldehyde group being positioned into the solvent.

**Figure 3:**
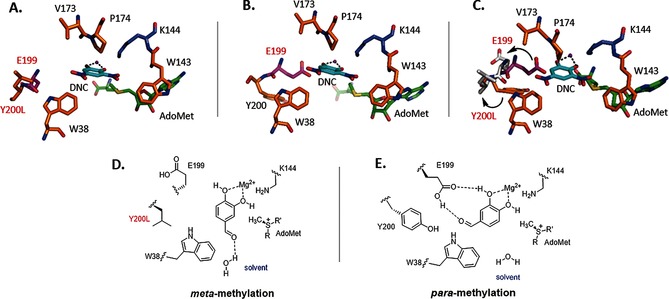
Crystal structures of COMT and proposed substrate binding modes for *para*‐ and *meta*‐methylation. Mg^2+^ (magenta sphere) is shown coordinated (dashed lines) to the catechol hydroxy groups. A) COMT mutant Y200L complexed with DNC, showing the Y200L mutation that causes the adjacent E199 residue to flip out of the active site and lose contact with the catechol. B) Crystal structure of WT COMT monomer complexed with DNC (PDB ID: 1VID), with E199 in close proximity to the R group of bound substrates. C) Overlay of the Y200L and WT structures, showing the altered positions of Y200L and E199 in the mutant (grey). D) Substrate orientation for *meta*‐methylation in Y200L. With E199 unable to hydrogen bond with the substrate aldehyde, the aldehyde group preferentially associates with the solvent, which positions the *meta*‐hydroxy group adjacent to the AdoMet sulphonium centre. E) Substrate orientation for *para*‐methylation in WT COMT, with the substrate aldehyde hydrogen bonding with E199, thereby orientating the catechol ring such that the *para*‐hydroxy group is presented to AdoMet.

Gel filtration chromatography showed two different oligomeric states for COMT (Figure S7). Previous literature^8^ has indicated the presence of monomeric and dimeric forms of COMT, which is consistent with the calibrated FPLC elution times that we observed (Figure S8). The catalytic properties of the monomeric and dimeric COMT have not been previously investigated. Accordingly, the two forms were separated and their activity and regioselectivity determined with **1 a**, **1 b**, and **1 d** (Table S4, Figure S9–S12). The WT monomer showed significantly lower *re* values of +31 % (**1 a**), +48 % (**1 b**), and +33 % (**1 d**) compared to +84 % (**1 a**), +59 % (**1 b**), +87 % (**1 d**) for the dimer (Table S4). The *re* values for the reaction catalyzed by the nickel‐purified COMT (Figure 2), which exists as a mixture of monomer and dimer, lie between those of the monomeric and dimeric forms. In contrast, both oligomeric forms of Y200L showed high *re* values with **1 a** (ca +90 %; Figure S12). A crystal structure of dimeric WT COMT with bound AdoMet and DNC was solved to 1.6 Å resolution (Figure S13, PDB ID: 5FHQ). Unlike with monomeric WT COMT, the β7 strand exchange between the two subunits that occurs in the dimer maintains the overall Rossman fold tertiary structure; however, E199 and Y200 lie on the β6–β7 interconnecting loop, which is pulled out of the active site as a result of domain swapping, thus leading to loss of the E199–substrate interaction. Since monomeric Y200L is as regioselective as the dimeric forms (ca. +90 % *re*), we suggest, based on the structural data for the Y200L and WT dimers and the regioselectivity assay data for both oligomeric forms, that loss of *para*‐methylation is largely due to movement of E199 out of the active site, as a consequence either of mutagenesis (Y200L) or protein dimerization and domain swapping (WT). The presence of two structural forms of COMT, each with contrasting activity and regioselectivity, is unusual. Whilst it is not entirely clear, we suggest that there may be two possible explanations to account for our observation. The physiologically relevant form of COMT in vivo may be the dimer, since mainly *meta*‐methylated catecholamine metabolites are found in urine and liver samples.^9^ Indeed, the first evidence to suggest that the catabolic pathway of catecholamines involves both *meta*  and significant levels of *para* products is based on in vitro data with monomeric COMT.^10^ However, another explanation is that the hexa‐His tag that we introduced at the C‐terminus may have contributed to domain swapping and dimerization.

Finally, we demonstrated potential applications for our improved COMT variants by regioselectively alkylating **1 a** by using ethyl, allyl, and benzyl AdoMet analogues generated enzymatically or synthetically (Figure 4, Figure S14).^11^ Products **4 a**–**c** were obtained with *re* values of approximately +90 % with Y200L compared to +33–45 % with WT COMT. Ethyl vanillin **4 a**, a high‐value food and flavor agent, was subsequently produced in 58 % yield of isolated product in a one‐pot tandem enzyme transformation from **1 a**, ethionine (**6**), and ATP, using a mutant human methionine‐adenosyltransferase hMAT2A (I322V)11b,11d and COMT Y200L (see Figure 4, Figure S14). Allyl vanillin **4 b** and benzyl vanillin **4 c** were obtained in 72 % and 63 % yields of isolated product, respectively. Although the yields for alkylation of DHBAL were good, further engineering of the regioselective COMT variants to improve activity with AdoMet analogues would be desirable for increased productivity.

**Figure 4:**
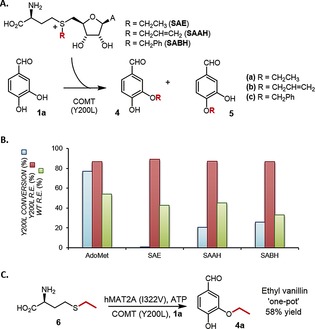
A) Transfer of alkyl groups from AdoMet analogues to **1 a** by using COMT. B) Comparison of conversions by Y200L COMT with AdoMet analogues after 20 min, and comparison of regioselectivity of Y200L versus WT COMT with AdoMet analogues. C) Enzymatic synthesis of ethyl vanillin (**4 a**) from ethionine.

In conclusion, COMT was engineered to produce regiocomplementary mutants that deliver alkylated catechols in significant regioisomeric excess. Mutants W38D and Y200L gave a high *re* in favor of the *meta* product with **1 a** and **1 d**, whilst W38R gave an *re* in favor of the *para* products with **1 b** and **1 c**. Moreover, K144 mutants retained significant activity and showed *para*‐selectivity with **1 a** and **1 d**. The X‐ray crystal structures of dimeric Y200L and WT COMT revealed that a key residue (E199) had been flipped out of the active site. We rationalize that in the WT monomeric enzyme, E199 can form a hydrogen bond with the aldehyde group of **1 a**, thereby leading to some *para*‐methylation, whereas in dimeric Y200L and WT COMT the absence of E199 from the active site results in the aldehyde group orientating towards the solvent, thereby leading to *meta*‐methylation. However, the *re* value for Y200L monomer (ca. +90 %) suggests that in the absence of dimerization, the Y200L mutation alone is sufficient to cause loss of E199 interaction with the substrate. Active‐site modifications thus appear to cause significant shifts in regioselectivity independently of the enzyme oligomeric state. The improved *meta*‐selective COMT mutants could be used to enhance production of vanillin in yeast or *E. coli*.^3^, ^4^ These COMT variants also accept AdoMet analogues to deliver valuable regioselectively alkylated catechols, including ethyl vanillin, which was prepared in a one‐pot manner from ATP, DHBAL, and ethionine.

## Supporting information

As a service to our authors and readers, this journal provides supporting information supplied by the authors. Such materials are peer reviewed and may be re‐organized for online delivery, but are not copy‐edited or typeset. Technical support issues arising from supporting information (other than missing files) should be addressed to the authors.

Supplementary

## References

[1a] U. T. Bornscheuer , G. W. Huisman , R. J. Kazlauskas , S. Lutz , J. C. Moore , K. Robins , Nature 2012, 485, 185–194; 22575958 10.1038/nature11117

[1b] K. E. Jaeger , T. Eggert , Curr. Opin. Biotechnol. 2004, 15, 305–313; 15358000 10.1016/j.copbio.2004.06.007

[1c] R. N. Patel , ACS Catal. 2011, 1, 1056–1074;

[1d] M. T. Reetz , J. Am. Chem. Soc. 2013, 135, 12480–12496; 23930719 10.1021/ja405051f

[1e] L. Cipolla , M. Lotti , L. De Gioia , F. Nicotra , J. Carbohydr. Chem. 2003, 22, 631–644;

[1f] K. Pollmann , V. Wray , H. J. Hecht , D. H. Pieper , Microbiology 2003, 149, 903–913; 12686633 10.1099/mic.0.26054-0

[1g] S. Kumar , E. E. Scott , H. Liu , J. R. Halpert , J. Biol. Chem. 2003, 278, 17178–17184. 12609983 10.1074/jbc.M212515200

[2a] E. K. Lim , D. A. Ashford , B. Hou , R. G. Jackson , D. J. Bowles , Biotechnol. Bioeng. 2004, 87, 623–631; 15352060 10.1002/bit.20154

[2b] J. Rehdorf , M. D. Mihovilovic , U. T. Bornscheuer , Angew. Chem. Int. Ed. 2010, 49, 4506–4508; 10.1002/anie.201000511 20455228

[2c] C. Wuensch , S. M. Glueck , J. Gross , D. Koszelewski , M. Schober , K. Faber , Org. Lett. 2012, 14, 1974–1977. 22471935 10.1021/ol300385k PMC3593611

[3] E. H. Hansen , B. L. Møller , G. R. Kock , C. M. Bünner , C. Kristensen , O. R. Jensen , F. T. Okkels , C. E. Olsen , M. S. Motawia , J. Hansen , Appl. Environ. Microbiol. 2009, 75, 2765–2774. 19286778 10.1128/AEM.02681-08 PMC2681717

[4a] K. Li , J. W. Frost , J. Am. Chem. Soc. 1998, 120, 10545–10546;

[4b] A. R. Brochado , C. Matos , B. L. Møller , J. Hansen , U. H. Mortensen , K. R. Patil , Microb. Cell Fact. 2010, 9, 84. 21059201 10.1186/1475-2859-9-84 PMC2992047

[5a] J. Vidgren , L. A. Svensson , A. Liljas , Nature 1994, 368, 354–358; 8127373 10.1038/368354a0

[5b] P. N. Palma , M. L. Rodrigues , M. Archer , M. J. Bonifa , A. I. Loureiro , D. A. Learmonth , M. A. Carrondo , Mol. Pharmacol. 2006, 70, 143–153; 16618795 10.1124/mol.106.023119

[5c] D. Tsao , S. Liu , N. V. Dokholyan , Chem. Phys. Lett. 2011, 506, 135–138. 21731105 10.1016/j.cplett.2011.03.048 PMC3125089

[6a] C. R. Creveling , N. Dalgard , H. Shimizu , J. W. Daly , Mol. Pharmacol. 1970, 6, 691–696; 5497718

[6b] C. R. Creveling , N. Morris , H. Shimizu , H. H. Ong , J. Daly , Mol. Pharmacol. 1972, 8, 398–409; 4340872

[6c] D. R. Thakker , C. Boehlerts , K. L. Kirks , R. Antkowiak , C. R. Creveling , J. Biol. Chem. 1986, 261, 178–184. 3753600

[7] J. Zhang , J. P. Klinman , J. Am. Chem. Soc. 2011, 133, 17134–17137. 21958159 10.1021/ja207467d PMC3219439

[8] M. Ellermann , R. Paulini , R. Jakob-Roetne , C. Lerner , E. Borroni , D. Roth , A. Ehler , W. B. Schweizer , D. Schlatter , M. G. Rudolph , et al., Chem. Eur. J. 2011, 17, 6369–6381. 21538606 10.1002/chem.201003648

[9a] J. Axelrod , R. Tomchick , J. Biol. Chem. 1958, 233, 702–705; 13575440

[9b] J. Axelrod , J. K. Inscoe , S. Senoh , B. Witkop , Biochim. Biophys. Acta 1958, 27, 210–211; 13510275 10.1016/0006-3002(58)90316-0

[9c] J. W. Daly , J. Axelrod , B. Witkop , J. Biol. Chem. 1960, 235, 1155–1159. 13813871

[10] S. Senoh , J. Daly , J. Axelrod , B. Witkop , J. Am. Chem. Soc. 1959, 81, 6240–6245.

[11a] C. Dalhoff , G. Lukinavičius , S. Klimasăuskas , E. Weinhold , Nat. Chem. Biol. 2006, 2, 31–32; 16408089 10.1038/nchembio754

[11b] R. Wang , K. Islam , Y. Liu , W. Zheng , H. Tang , N. Lailler , G. Blum , H. Deng , M. Luo , J. Am. Chem. Soc. 2013, 135, 1048–1056; 23244065 10.1021/ja309412s PMC3582175

[11c] S. Singh , J. Zhang , T. D. Huber , M. Sunkara , K. Hurley , R. D. Goff , G. Wang , W. Zhang , C. Liu , J. Rohr , et al., Angew. Chem. Int. Ed. 2014, 53, 3965–3969; 10.1002/anie.201308272 PMC4076696 24616228

[11d] B. J. C. Law , A.-W. Struck , M. R. Bennett , B. Wilkinson , J. Micklefield , Chem. Sci. 2015, 6, 2885–2892. 10.1039/c5sc00164a PMC5729408 29403635

