# Supplementary material for: Effects of Active‐Site Modification and Quaternary Structure on the Regioselectivity of Catechol‐O‐Methyltransferase
Source: Angew Chem Int Ed Engl. 2016 Jan 21;55(8):2683–7. doi: 10.1002/anie.201508287 (PMC4770447; doi:10.1002/anie.201508287)
Supplement: Supplementary file 1 — Supplementary [file ANIE-55-2683-s001.pdf]

Angewandte Chemie
Eine Zeitschrift der Gesellschaft Deutscher Chemiker

Supporting Information

Effects of Active-Site Modification and Quaternary Structure on the Regioselectivity of Catechol-*O*-Methyltransferase

*Brian J. C. Law, Matthew R. Bennett, Mark L. Thompson, Colin Levy, Sarah A. Shepherd, David Leys, and Jason Micklefield**

anie_201508287_sm_miscellaneous_information.pdf

Supporting Information

Table of Contents

pp. S3-S6.	Experimental methods
pp. S6-S9.	Synthesis of reaction standards
pp. S10-S30.	Supplementary figures and tables
	Figure S1. Biosynthesis of vanillin from glucose
	Figure S2. Methylated catechols in APIs
	Figure S3. Irreversibility of COMT-catalysed methylation
	Table S1. Activity and regioselectivity of COMT mutants
	Figure S4. Activity and regioselectivity of COMT mutants
	Table S2. Kinetic parameters for selected COMT enzymes
	Figure S5. pH profile of WT and K144A COMT
	Table S3. Crystallography data for WT dimeric and Y200L dimeric COMT
	Figure S6. Preliminary X-ray crystal structure of Y200L COMT with DHBAL and SAH
	Figure S7. Gel filtration chromatogram for COMT monomer and dimer
	Figure S8. Calibration for gel filtration chromatography
	Table S4. WT monomer and dimer activity and regioselectivity
	Figure S9. Oligomeric forms of COMT in solution
	Figure S10. Stability of WT COMT under dilution and typical reaction conditions
	Figure S11. Stability of WT and Y200L monomeric and dimeric forms
	Figure S12. Regioselectivity of WT and Y200L monomeric and dimeric forms
	Figure S13. X-ray crystal structure of dimeric COMT with DNC and AdoMet bound
	Figure S14. AdoMet analogue assays
	Table S5. Primers used for COMT mutagenesis
	Figure S15. SDS PAGE of Ni-NTA purification of COMT
	Figure S16. SDS PAGE of COMT anion exchange and gel filtration purification
	Figure S17. HPLC analyses of COMT assays with authentic standards
	Figure S18. Calibration curves for substrates **1a-d** and products **2a-d** and **3a-d**
	Figure S19. ^{1}H NMR of enzymatic **4a**
	Figure S20. HPLC chromatogram of **4a**
	Figure S21. ^{1}H NMR of enzymatic **4b**
	Figure S22. HPLC chromatogram of **4b**
	Figure S23. ^{1}H NMR of enzymatic **4c**
	Figure S24. HPLC chromatogram of **4c**
p. S31.	Supplementary references

Experimental

We thank Professor Nigel Scrutton (University of Manchester) for the plasmid pET21b-COMT, and Professor Udo Oppermann (Oxford) for the plasmid pNIC28-bsa4-hMAT2A.

Site-directed mutagenesis of rat soluble-COMT (S-COMT). The expression construct pET21b-COMT carrying an *E. coli* codon-optimised rat soluble-COMT gene with a C-terminal hexa-His tag was kindly provided by Professor Nigel Scrutton (University of Manchester). Mutagenic primers carrying codon changes at the W38, K144, V173, E199 and Y200 positions (Table S5) were used to introduce targeted mutations of these active site residues to one of a diverse range of amino acids (Ala, Asp, Glu, Arg, Lys, His, Leu, Phe, Tyr, Trp), using standard PCR techniques. The parental wild-type DNA was digested with the addition of *Dpn*I restriction endonuclease. The newly synthesised mutant DNA was subsequently transformed into *E. coli* DH5α chemically competent cells for plasmid amplification, extracted using a Qiagen miniprep spin kit and verified by nucleotide sequencing (GATC Biotech).

Overproduction and purification of rat S-COMT. The wild-type and mutant COMT expression plasmids were transformed into *E. coli* BL21 (DE3) chemically competent cells for protein production. The cells were maintained on LB agar containing ampicillin (50 µg/mL). Single colonies were used to inoculate LB seeder cultures (containing ampicillin, 50 µg/mL) and cultivated at 37 °C, 18 h, 190 rpm agitation. The seeder cultures were diluted 100-fold into fresh LB (ampicillin, 50 µg/mL) and cultivated at 37 °C, 190 rpm agitation until an OD_{600} of 0.6-0.8 was reached. Protein production was induced with the addition of isopropyl β-D-1-thiogalactopyranoside (IPTG, 0.5 mM) and the cells were cultivated for a further 3 h at 37 °C, 190 rpm agitation, before the cells were harvested by centrifugation (1600 *x g*, 10 min, 4 °C). The cell pellets were resuspended (15 mL buffer per pellet from 800 mL culture) in lysis buffer (50 mM potassium phosphate buffer pH 7.4, 300 mM NaCl, 10 mM imidazole) and lysed by sonication. The cell lysates were clarified by centrifugation (11,000 *x g*, 4 °C, 45 min) and the soluble lysate loaded over a Ni-NTA gravity flow column. The column was washed first with lysis buffer containing 30 mM imidazole (7 column volumes, CV) followed by buffer containing 60 mM imidazole (5 CV). The COMT protein was eluted with lysis buffer containing 250 mM imidazole (5 CV) and simultaneously dialysed and concentrated into storage buffer (50mM potassium phosphate pH 7.4, 300 mM NaCl) to approximately 1 mL volume using a 10,000 MWCO Vivaspin 20 centricon (Sartorius Stedim Biotech). All fractions were analysed by SDS-PAGE for purity (Fig. S15). Glycerol was added to 10% (v/v) and the enzyme stored at -80 °C. Enzymes purified in this manner were appropriately pure for enzymatic assays. For crystallography trials, the protein was further purified by anion exchange and gel filtration chromatography (Figs. S7 & S16).

Anion exchange and gel filtration chromatography of COMT for crystallography. Anion exchange chromatography was carried out using an ÄKTA prime FPLC with a HiTrap™ Q HP anion exchange column (GE Healthcare). The COMT protein eluate from the Ni-NTA purification of COMT was diluted into anion exchange buffer (50 mM Tris-HCl, pH 8) to reduce the NaCl concentration to < 20 mM and loaded onto the anion exchange column. The column was washed with Tris buffer containing 50 mM NaCl until the UV absorbance stabilised, whereupon an isocratic gradient (100%) of anion exchange elution buffer (50 mM Tris-HCl pH 8, 1 M NaCl) was applied to elute the protein. The fractions collected were analysed by SDS-PAGE.

Gel filtration chromatography was carried out using a Superdex™ 75 10/300 GL column (GE Healthcare) on an ÄKTApurifier FPLC system. Fractions from the anion exchange purification containing COMT were pooled and buffer exchanged into gel filtration buffer (50 mM Tris-HCl pH 7.5, 50 mM NaCl, 10 mM DTT, 2 mM $MgCl_2$) under volume reduction using a Vivaspin 20 centricon (10,000 MWCO). The protein concentrate (approximately 6 mg/mL) was loaded onto the column in 0.5 mL injections. An isocratic gradient of 100% gel filtration buffer was used, at a flow rate of 1 mL/min. Fractions were collected in a 96-deep well block and analysed by SDS-PAGE.

Enzyme activity and regioselectivity assays. Wild-type and mutant COMT enzymes were assayed against the substrates DHBAL (**1a**), DHBA (**1b**), DHPA (**1c**) and NOC (**1d**) as follows: reaction mixtures contained 1 mM DTT, 1 mM SAM, 3 mM $MgCl_2$, 0.5 mM substrate, 5 µM COMT, in phosphate buffer, pH 7.4. The reactions were incubated at 37 °C, with 800 rpm agitation for 20 min in an Eppendorf Thermomixer and subsequently quenched with an equal volume of methanol. The protein precipitate was removed by centrifugation and the assays analysed by C_{18} RP-HPLC using a Shimadzu Prominence UFLC XR HPLC system: (Phenomenex Kinetex C_{18} 5 µm 4.6 x

250 mm column, flow rate 1 mL/min, wavelength 278 nm (**1a-c**), 340 nm (**1d**)); gradient (**1a-c**) 0-2 min 5% B-20% B, 2-11 min 20% B-23% B, 11-13 min 95% B, 13-15 min 5% B; gradient (**1d**) 0-2 min 5% B-20% B, 2-11 min 20% B-70% B, 11-13 min 95% B, 13-15 min 5% B. Mobile phase A consisted of H_2O + 0.05% TFA, mobile phase B consisted of acetonitrile + 0.05% TFA. The substrate, *meta*- and *para*-regioisomer methylated peaks for each of **1-3**(**a-d**) were compared with authentic standards (Sigma Aldrich UK) as shown in (Fig. S17). Calibration curves (Fig. S18) were made for compounds **1-3**(**a-d**) and the HPLC peak areas adjusted accordingly to account for differences in extinction coefficients. The relative activities were calculated as the percentage of product formed from the starting material, whilst regioselectivity was calculated as the regiomeric excess of the *meta*-regiosiomer over the *para*-regioisomer, with negative values indicating preference for *para*-methylation. Activities and regioselectivities for the full set of mutants are shown in (Fig. S4 & Table S1).

Michaelis-Menten enzyme kinetics. To determine the kinetic constants of wild-type and mutant COMT, enzyme (0.2-0.5 μM) was assayed against a range of substrate concentrations (2-5000 μM) with the initial rate of reaction measured by monitoring substrate conversion over several time points. The rates were plotted against substrate concentrations using Sigmaplot 12.0 and the K_m and k_{cat} constants generated from the resulting Michaelis-Menten plot.

Crystallogenesis. The sitting drop vapour diffusion technique was used to grow crystals of both wild type COMT dimer and the Y200L mutant. The purified protein was collected, diluted (1 μM) and mixed with SAM (10 μM) and DNC (10 μM) overnight at 4 ˚C through gentle rocking. The COMT proteins were then concentrated to a final concentration of 10 mg/mL with bound DNC and SAM ligands in a 20 mM Tris buffer (with 300 mM NaCl) at pH 7.4. The wild type COMT crystals were grown by mixing 200 nL of protein with an equal volume of reservoir solution comprising (0.09 M [0.2 M sodium formate; 0.2 M ammonium acetate; 0.2 M sodium citrate tribasic dihydrate; 0.2 M sodium potassium tartrate tetrahydrate; 0.2 M sodium oxamate] 0.1 M Tris / Bicine pH 8.5, 50% [40% v/v PEG 500* MME; 20 % w/v PEG 20000] Morpheus HT96 condition G9). The Y200L crystals were grown in a similar manner but utilizing a reservoir solution comprising (0.09 M [0.3 M sodium fluoride; 0.3 M sodium bromide; 0.3 M sodium iodide], 0.1 M Tris / Bicine pH 8.5, 50% [40% v/v PEG 500* MME; 20 % w/v PEG 20000] Morpheus HT96 condition B9). All drops were set using a Mosquito (TTP) pipetting robot and incubated at a temperature of 4 °C for 16 hours. Following incubation single crystals suitable for X-Ray analysis were flash frozen by plunge freezing in LN2.

Crystallography data collection and structure determination. Data were collected from single cryo frozen crystals at Diamond Light Source, full details of data and refinement statistics are presented in Table S2. The data was scaled and integrated using Xia2 [S1] and the structures subsequently solved by molecular replacement in Phaser. [S2] All models were subsequently completed and refined using iterative cycles of rebuilding and refinement in COOT [S3] and Phenix.refine.[S4] Validation with Molprobity was integrated into the iterative rebuilding and refinement cycle.[S5] Final models with R and Rf of 15.5 & 18.8 for the WT dimer structure and 15.9 and 17.8 for the Y200L dimer structure with DNC have been deposited with the protein data bank, accession codes 5FHQ, 5FHR.

AdoMet analogue assays (Fig. 4B). Expression and purification of hMAT2A (I322V), and preparation of the enzymatically-generated AdoMet analogues were as previously described.[11d] Wild-type and mutant COMT enzymes were assayed against the substrate DHBAL **1a** as follows: reaction mixtures containing 1 mM DTT, 0.2 mM AdoMet analogue (SAE, SAAH or SABH), 3 mM $MgCl_2$, 0.1 mM substrate, 45 μM COMT, in phosphate buffer pH 7.4, were incubated at 37 °C with 800 rpm agitation for 3 hours. The reactions were subsequently quenched with an equal volume of methanol, and the protein precipitate removed by centrifugation. The assays were analysed for conversions and r.e.s (fig. 4B) by C_{18} RP-HPLC using an Agilent 1260 Infinity HPLC system: (Phenomenex Kinetex C_{18} 5 μm 4.6 x 150 mm column, flow rate 1 mL/min; wavelength 278 nm), gradient 0-2 min 5% B, 1-3 min 5-20% B, 3-4 min 20-50% B, 4-9 min 50-70% B, 9-11 min 70% B, 11.1-13.0 min 5% B. Mobile phase A consisted of 5 mM potassium phosphate buffer, mobile phase B consisted of acetonitrile.

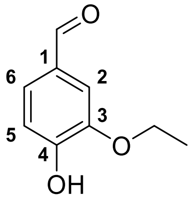

4a

Ethyl vanillin 4a was prepared using a coupled enzyme reaction with hMAT2A (I322V) and COMT (Y200L) as follows: the reaction mixture containing 1 mM DTT, 3 mM ATP, 3 mM ethionine, 3 mM $MgCl_2$, 0.5 mM DHBAL **1a**, 180 µM COMT (Y200L), 25 µM hMAT2A (I322V) in 20 mM phosphate buffer pH 7.4, was incubated at 37 °C with 800 rpm agitation for 16 hours. The reaction was quenched with aqueous formic acid (1%) and the protein precipitate was removed by centrifugation. The supernatant was purified using an Agilent BondElut Solid Phase Extraction cartridge followed by RP-HPLC using a Varian Pro Star HPLC system: (Phenomenex Kinetex C_{18} 5 µm 10 x 150 mm column, flow rate 5 mL/min, wavelength 278 nm), gradient 0-1 min 5% B, 1-2 min 5% B-35% B, 2-8 min 35% B-70% B, 8-10 min 95% B, 10-12 min 5% B. Mobile phase A consisted of H_2O + 0.05% TFA, mobile phase B consisted of acetonitrile + 0.05% TFA. 3-ethoxy-4-hydroxybenzaldehyde **4a** (1.8 mg, 58%) was isolated and shown to be identical to an authentic sample (Sigma Aldrich UK) by ^1H, HPLC and mass spectrometry. ^1H NMR (400 MHz, $CDCl_3$) δ 9.83 (s, 1H, CHO), 7.43 (d, *J* = 8.3 Hz, 1H, H6), 7.41 (s, 1H, H2), 7.06 (d, *J* = 8.3 Hz, 1H, H5), 6.21 (s, 1H, OH), 4.22 (q, *J* = 7.1 Hz, 2H, CH_2), 1.50 (t, *J* = 7.1 Hz, 3H, CH_3). ES^+ MS (%) 167.1 (100) $[M+H]^+$ (C_7H_6O expected mass 167.0708). ES^+MS (%) 167 (100) $[M+H]^+$, HRMS ES^+MS, mass calculated for $C_9H_{11}O_3^+$: 167.0708; found 167.0705.

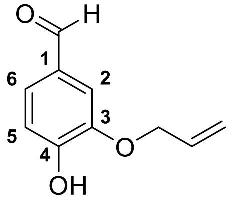

4b

Allyl vanillin (3-allyloxy-4-hydroxybenzaldehyde) 4b was prepared using a coupled enzyme reaction with hMAT2A (I322V) and COMT (Y200L) as follows: the reaction mixture containing 1 mM DTT, 2 mM *S*-allyl-homocysteine, 3 mM $MgCl_2$, 0.5 mM DHBAL **1a**, 45 µM COMT (Y200L), 5 µM hMAT2A (I322V) in 20 mM phosphate buffer pH 7.4, was incubated at 37 °C with 800 rpm agitation for 16 hours. The protein was precipitated and the product solubilised through addition of ethyl acetate (5 mL). The mixture was centrifuged (4500 *x g*, 20 min) and the ethyl acetate layer transferred to a new vessel. The remaining aqueous was further extracted (2 x 5 mL ethyl acetate) as described previously. The organic fractions were combined and dried under a stream of nitrogen. The resulting residue was dissolved in 60% aqueous acetonitrile. The solution was then purified by RP-HPLC using a Varian Pro Star HPLC system: (Phenomenex Kinetex C_{18} 5 µm 10 x 150 mm column, flow rate 5 mL/min, wavelength 278 nm), gradient 0-1 min 5% B, 1-2 min 5% B-35% B, 2-8 min 35% B-70% B, 8-10 min 95% B, 10.0-12 min 5% B. Mobile phase A consisted of H_2O + 0.05% TFA, mobile phase B consisted of acetonitrile + 0.05% TFA. 3-allyloxy-4-hydroxybenzaldehyde **4b** (14 mg, 72%) was isolated and shown to be identical to an authentic sample by ^1H, HPLC and mass spectrometry. ^1H (400MHz, $CDCl_3$) δ 9.82 (s, 1H, CHO), 7.45 – 7.42 (m, 2H, H2+H6), 7.06 (d, *J* = 8.0 Hz, 1H, H5), 6.30 (s, 1H, OH), 6.07 (ddt, *J* = 17.2, 10.3, 5.6 Hz, 1H, $CH_2\underline{CH}{=}CH_2$), 5.45 (ddt, *J* = 10.4, 1.4, 1.4 Hz, 1H, $CH_2CH{=}\underline{CH_2}$'), 5.37 (ddt, *J* = 10.4, 1.3, 1.3 Hz, 1H, $CH_2CH{=}\underline{CH_2}$), 4.69 (ddd, *J* = 5.6, 1.4, 1.4 Hz, 2H, $\underline{CH_2}CH{=}CH_2$), HRMS ES^+MS: mass calculated for $C_9H_{11}O_3^+$: 179.0703; found 179.0705.

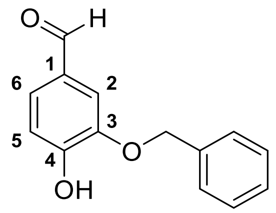

4c

Benzyl vanillin (3-benzoxy-4-hydroxybenzaldehyde) 4c was prepared using an enzyme reaction with COMT (Y200L) and the synthetic *S*-adenosyl-benzyl-homocysteine cofactor as follows: the reaction mixture containing 1 mM DTT, 2 mM *S*-adenosyl-benzyl-homocysteine, 3 mM $MgCl_2$, 0.5 mM DHBAL **1a**, 170 µM COMT (Y200L) in 20 mM phosphate buffer pH 7.4, was incubated at 37 °C with 800 rpm agitation for 16 hours. The protein was precipitated and the product solubilised through addition of ethyl acetate (5 mL). The mixture was centrifuged (4500 *x g*, 20 min) and the ethyl acetate layer transferred to a new vessel. The remaining aqueous was further extracted (2 x 5 mL ethyl acetate) as described previously. The organic fractions were combined and dried under a stream of nitrogen. The resulting residue was dissolved in 60% aqueous acetonitrile. The solution was then purified by RP-HPLC using a Varian Pro Star HPLC system: (Phenomenex Kinetex C_{18} 5 µm 10 x 150 mm column, flow rate 5 mL/min, wavelength 278 nm), gradient 0-1 min 5% B, 1-2 min 5% B-35% B, 2-8 min 35% B-70% B, 8-10 min 95% B, 10-12 min 5% B. Mobile phase A consisted of H_2O + 0.05% TFA, mobile phase B consisted of acetonitrile + 0.05% TFA. 3-benzyloxy-4-hydroxybenzaldehyde **4c** (2.6 mg, 63%) was isolated and shown to be identical to an authentic sample by ^{1}H, HPLC and mass spectrometry. ^{1}H (400MHz, CD_3CN) δ 9.77 (s, 1H, CHO), 7.52-7.35 (m, 7H, Ph, H6 & H5), 7.01 (d, *J* = 8.0 Hz, 1H, H5), 5.23 (s, 2H, H1'), HRMS ES^+MS, mass calculated for $C_{14}H_{13}O_3^+$: 229.0859; found 229.0861.

Synthesis of reaction standards

Commercial standards. 3,4-dihydroxybenzaldehyde (DHBAL, **1a**), vanillin (**2a**), isovanillin (**3a**), 3-ethoxy-4-hydroxybenzaldehyde (ethyl vanillin, **4a**), 3,4-dihydroxybenzoic acid (DHBA, **1b**), vanillic acid (**2b**), isovanillic acid (**3b**), 3,4-dihydroxyphenylacetic acid (DHPA, **1c**), homovanillic acid (**2c**), isohomovanillic acid (**3c**), 4-nitrocatechol (NOC, **1d**), 4-nitroguaiacol (**2d**), 2-methoxy-5-nitrophenol (**3d**) were purchased as authentic standards from Sigma Aldrich UK.

The synthesis of reaction standards was carried out by adapting a procedure previously reported by Reitz *et al* for the preparation of 3-allyloxy-4-hydroxybenzaldehyde and 4-allyloxy-3-hydroxybenzaldehyde.[S6]

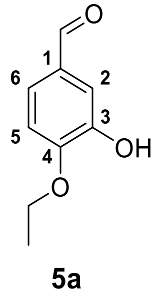

5a

4-ethoxy-3-hydroxybenzaldehyde (5a). A suspension of 3,4-dihydroxybenzaldehyde (**1a)** (3.1 g, 23 mmol), acetone (100 mL), potassium carbonate (3.1 g, 23 mmol) and ethyl iodide (3.6 g, 1.8 mL, 23 mmol) was stirred for 5 hours at 60 ˚C. After 4 hours the suspension was filtered and the acetone removed under reduced pressure. The resulting residue was dissolved in diethyl ether (50 mL) and mixed with H_2O. The mixture was acidified to pH 3 with aqueous sulphuric acid (6.0 M). The layers were then separated and the aqueous extracts were further extracted

with diethyl ether (2 x 50 mL). The organic fractions were combined and dried over magnesium sulphate. The solvent was removed under reduced pressure and the crude product was purified by flash chromatography (80:20:1, hexane/ethyl acetate/acetic acid) to give 4-ethoxy-3-hydroxybenzaldehyde (0.73 g, 19%). ^{1}H NMR (400 MHz, $CDCl_3$) δ 9.84 (s, 1H, CHO), 7.45 (d, *J* = 1.9 Hz, 1H, H2), 7.42 (dd, *J* = 1.9, 8.1 Hz, 1H, H6), 6.95 (d, *J* = 8.1 Hz, 1H, H5), 5.84 (s, 1H, OH), 4.22 (q, *J* = 7.1 Hz, 2H, CH_2), 1.52 (t, *J* = 7.1 Hz, 3H, CH_3), ^{13}C NMR (100 MHz, $CDCl_3$) δ 191.1 (CHO), 150.7 (C4), 146.3 (C3), 130.2 (C1), 124.7 (C6), 114.2 (C2), 111.0 (C5), 65.0 (CH_2), 14.8 (CH_3). IR (neat) $\tilde{\upsilon}$ = 3182, 2848, 1664, 1582, 1506, 1457, 1393, 1273 cm^{-1}. ES$^-$ MS (%) 165.0 (40) $[M-H]^-$, 137.0 $[M-C_2H_5]^-$ (100) (C_7H_6O expected mass 165.0557), ES$^+$MS (%) 167.0 (100) $[M+H]^+$, HRMS ES$^+$MS, mass calculated for $C_9H_{11}O_3^+$: 167.0708; found 167.0703.

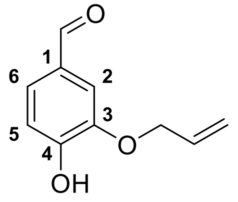

4b

3-allyloxy-4-hydroxybenzaldehyde (4b). 60% sodium hydride in mineral oil (3.8 g, 81 mmol) was washed free of oil with four portions of dry hexane (30 mL) under nitrogen. Dry DMSO (60 mL) was then added and the mixture was then cooled to 0 ˚C with stirring. A solution of 3,4-dihydroxybenzaldehyde (**1a**) (5.5 g, 40 mmol) in dry DMSO (20 mL) was then added dropwise. The suspension was stirred until no solid was visible, whereupon a solution of allyl bromide (4.8 g, 3.5 mL, 40 mmol) in dry DMSO (20 mL) was added dropwise. The ice bath was then removed and the solution was left to reach room temperature whilst stirring overnight. The solution was then added to ice cool water (100 mL) and acidified with aqueous HCl (1.0 M). The product was then extracted with ethyl acetate (3 x 100 mL). The organic layers were combined, washed with brine and dried over magnesium sulphate. The ethyl acetate was then removed under reduced pressure. The product was then purified through flash chromatography using hexane/ethyl acetate/acetic acid (80:20:1) as the eluant to give 3-allyloxy-4-hydroxybenzaldehyde (0.90 g, 13%). ^{1}H (400MHz, $CDCl_3$) δ 9.81 (s, 1H, CHO), 7.45 – 7.38 (m, 2H, H2+H6), 7.06 (d, *J* = 8.0 Hz, 1H, H5), 6.36 (s, 1H, OH), 6.08 (ddt, *J* = 17.2, 10.8, 5.6 Hz, 1H, $CH_2\underline{CH}=CH_2$), 5.46 (ddt, *J* = 17.2, 2.8, 1.3 Hz, 1H, $CH_2CH=\underline{CH_2}$’), 5.36 (ddt, *J* = 10.8, 2.8, 1.3 Hz, 1H, $CH_2CH=\underline{CH_2}$), 4.69 (ddd, *J* = 5.6, 1.3, 1.3 Hz, 2H, $\underline{CH_2}CH=CH_2$), ^{13}C (100MHz, $CDCl_3$) δ 191.3 (CHO), 153.3 (C4), 146.5 (C3), 132.4 ($CH_2\underline{CH}=CH_2$), 130.2 (C1), 128.0 (C6), 119.6 ($CH_2CH=\underline{CH_2}$), 115.1 (C5), 110.6 (C2), 70.4 ($\underline{CH_2}CH=CH_2$). ES$^-$ MS (%) 177 (60) $[M-H]^-$, 136 $[M-C_3H_5]^-$ (100), ES$^+$MS (%) 179 (100) $[M+H]^+$.

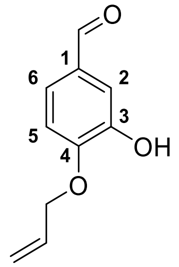

5b

4-allyloxy-3-hydroxybenzaldehyde (5b). A suspension of 3,4-dihydroxybenzaldehyde (**1a)** (3.1 g, 23 mmol), acetone (100 mL), potassium carbonate (3.1 g, 23 mmol) and allyl bromide (2.8 g, 2 mL, 23 mmol) was stirred for 5 hours at 60 ˚C. After 4 hours the suspension was filtered and the acetone removed under reduced pressure. The resulting residue was dissolved in diethyl ether (50 mL) mixed with H_2O and acidified to pH 3 with aqueous sulphuric acid (6.0 M). The layers were then separated and the aqueous extracts were further extracted with diethyl

ether (2 x 50 mL). The organic fractions were combined and dried over magnesium sulphate. The solvent was removed under reduced pressure and the crude product was purified by flash chromatography (80:20:1, hexane/ ethyl acetate/acetic acid) to give 4-allyloxy-3-hydroxybenzaldehyde (1.1 g, 28%). ^{1}H (400MHz, $CDCl_3$) δ 9.84 (s, 1H, CHO), 7.42 (dd, J = 8.3, 2.0 Hz, 1H, H6), 7.47 (d, J = 2.0 Hz, 1H, H5), 6.98 (d, J = 8.3 Hz, 1H, H5), 6.29 (s, 1H, OH), 6.07 (ddt, J = 17.3, 10.6, 5.6 Hz, 1H, $CH_2\underline{CH}=CH_2$), 5.45 (ddt, J = 17.3, 2.8, 1.5 Hz, 1H, $CH_2CH=\underline{CH_2}$), 5.38 (ddt, J = 10.6, 2.8, 1.5 Hz, 1H, $CH_2CH=\underline{CH_2}$), 4.71 (ddd, J = 5.6, 1.5, 1.5 Hz, 2H, $\underline{CH_2}CH=CH_2$), ^{13}C (100MHz, $CDCl_3$) δ 191.3 (CHO), 151.1 (C4), 146.3 (C3), 131.9 ($CH_2\underline{CH}=CH_2$), 130.6 (C1), 124.6 (C6), 119.2 ($CH_2CH=\underline{CH_2}$), 114.4 (C2), 111.5 (C5), 70.0 ($\underline{CH_2}CH=CH_2$),. IR (neat) $\tilde{\upsilon}$ = 3195, 1672, 1576, 1508, 1422, 1343, 1239 cm^{-1}. ES$^-$ MS (%) 177 (60) $[M-H]^-$, 136 $[M-C_3H_5]^-$ (100), ES$^+$MS (%) 179 (100) $[M+H]^+$, HRMS ES$^+$MS, mass calculated for $C_{10}H_{11}O_3$: 179.0708; found 179.0714.

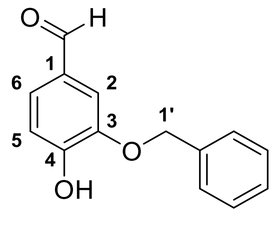

4c

3-benzoxy-4-hydroxybenzaldehyde (4c). 60% sodium hydride in mineral oil (3.8 g, 81 mmol) was washed free of oil with four portions of dry hexane (30 mL) under nitrogen. Dry DMSO (60 mL) was then added and the mixture was then cooled to 0 ˚C with stirring. A solution of 3,4-dihydroxybenzaldehyde (**1a**) (5.5 g, 40 mmol) in dry DMSO (20 mL) was then added dropwise. The suspension was stirred until no solid was visible, whereupon a solution of benzyl bromide (3.9 g, 2.7 mL, 23 mmol) in dry DMSO (20 mL) was added dropwise. The ice bath was then removed and the solution was left to reach room temperature whilst stirring overnight. The solution was then added to ice cool water (100 mL) and acidified with aqueous HCl (1.0 M). The product was then extracted with ethyl acetate (3 x 100 mL). The organic layers were combined, washed with brine and dried over magnesium sulphate. The ethyl acetate was then removed under reduced pressure. The product was then purified through flash chromatography using hexane/ethyl acetate/acetic acid (80:20:1) as the eluant to give 3-benzoxy-4-hydroxybenzaldehyde (0.10 g, 1%). ^{1}H (400MHz, CD_3CN) δ 9.77 (s, 1H, CHO), 7.52-7.37 (m, 7H, Ph, H6 & H5), 7.00 (d, J = 8.1 Hz, 1H, H5), 5.23 (s, 2H, H1'), ^{13}C (100MHz, CD_3CN) δ 191.8 (CHO), 153.5 (C4), 147.8 (C3), 137.6 (Ph), 130.9 (C1), 129.6 (Ph), 129.3 (Ph), 129.1 (Ph), 127.6 (C6), 116.3 (C5), 112.6 (C2), 71.7 ($\underline{CH_2}$-Ph), IR (neat) $\tilde{\upsilon}$ = 3242, 2855, 1665, 1587, 1511, 1437, 1386, 1274 cm^{-1}. ES$^+$MS (%) 229 (100) $[M+H]^+$, HRMS ES$^+$MS, mass calculated for $C_{14}H_{13}O_3^+$: 229.0859; found 229.0861.

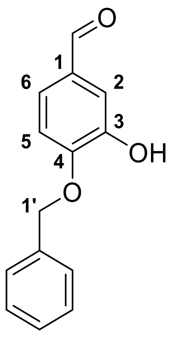

5c

3-hydroxy-4-benzoxybenzaldehyde (5c). A suspension of 3,4-dihydroxybenzaldehyde (**1a)** (3.1 g, 23 mmol), acetone (100 mL), potassium carbonate (3.1 g, 23 mmol) and benzyl bromide (3.9 g, 2.7 mL, 23 mmol) was stirred for 5 hours at 60 ˚C. After 4 hours the suspension was filtered and the acetone removed under reduced pressure.

The resulting residue was dissolved in diethyl ether (50 mL), mixed with H_2O and acidified to pH 3 with aqueous sulphuric acid (6.0 M). The layers were then separated and the aqueous layer further extracted with diethyl ether (2 x 50 mL). The organic fractions were combined and dried over magnesium sulphate. The solvent was removed under reduced pressure and the crude product was purified by flash chromatography (80:20:1, hexane/ethyl acetate/acetic acid) to give 4-benzoxy-3-hydroxybenzaldehyde (0.30 g, 7%). ^{1}H (400MHz, $CDCl_3$) δ 9.80 (s, 1H, CHO), 7.46 (d, *J* = 1.8 Hz, 1H, H2), 7.45-7.37 (m, 6H, Ph & H6), 7.04 (d, *J* = 8.3 Hz, 1H, H5), 6.12 (s, 1H, OH), 5.20 (s, 2H, $\underline{CH_2}$-Ph), ^{13}C (100MHz, $CDCl_3$) δ 191.2 (CHO), 151.0 (C4), 146.3 (C3), 135.2 (Ph), 130.6 (C1), 128.8 (Ph), 128.7 (Ph), 127.8 (Ph) 124.3 (C6) 114.0 (C2), 111.1 (C5), 71.2 ($\underline{CH_2}$-Ph), IR (neat) $\tilde{\upsilon}$ = 3178, 2869, 1670, 1576, 1510, 1453, 1388, 1280 cm^{-1}. ES$^-$MS (%) 227 (60) $[M-H]^-$, 136 $[M-C_3H_5]^-$ (100), ES$^+$MS (%) 229 (100) $[M+H]^+$, HRMS ES$^+$MS, mass calculated for $C_{14}H_{13}O_3^+$: 229.0859; found 229.0854.

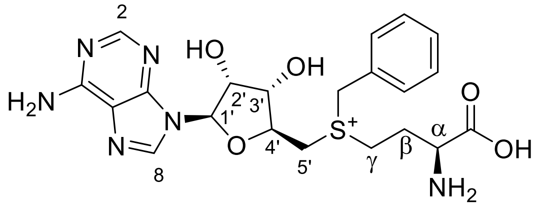

***S*-adenosylbenzylhomocysteine (SABH).** The synthesis of Dalhoff *et al* for *S*-adenosylallylhomocysteine was used to synthesise *S*-adenosylbenzylhomocysteine.[11a] A 1:1 mix of acetic acid and formic acid (3.0 mL) was added to SAH (20 mg, 52 µmol). The mixture was stirred on ice at 0 °C until dissolved. To this solution benzyl bromide (190 µL, 60 equivalents, and 3.1 mmol) was slowly added by dropwise addition. The ice bath was removed and the solution was left stirring for four days. Ice cold distilled water (30 mL) was then added and the resultant solution was washed with diethyl ether (3 x 5 mL). The aqueous layer was then lyophilized and purified by preparative RP-HPLC to give *S*-adenosylbenzylhomocysteine (22 mg, 46 µmol, 89 %). ^{1}H (400MHz, D_2O) δ 8.15 (s, 1H, H2), 8.10 (s, 1H, H8), 7.20 (m, 5H, Ph), 5.94 (d, *J* = 3.5, 1H, H1′), 4.63 (m, 1H, H2′), 4.54 (m, 1H, H3′), 4.39 (m, 1H, H4′), 3.85 (d, *J* = 6.8 Hz, 1H, H5b′) 3.77 (d, *J* = 6.8 Hz, 1H, H5a′), 3.71 (t, *J* = 6.8 Hz, 1H, Hα), 3.59 (m, 1H, Hγb), 3.41 (m, 1H, Hγa), 2.64 (s, $\underline{CH_2}$-Ph), 2.24 (q, *J* = 7.9 Hz, 2H, Hβ). MALDI (relative intensity): 475.2 $[M]^+$ (100) HRMS ES$^+$MS, mass calculated for $C_{21}H_{27}N_6O_5S^+$: 475.1758; found 475.1768.

Supplementary figures and tables

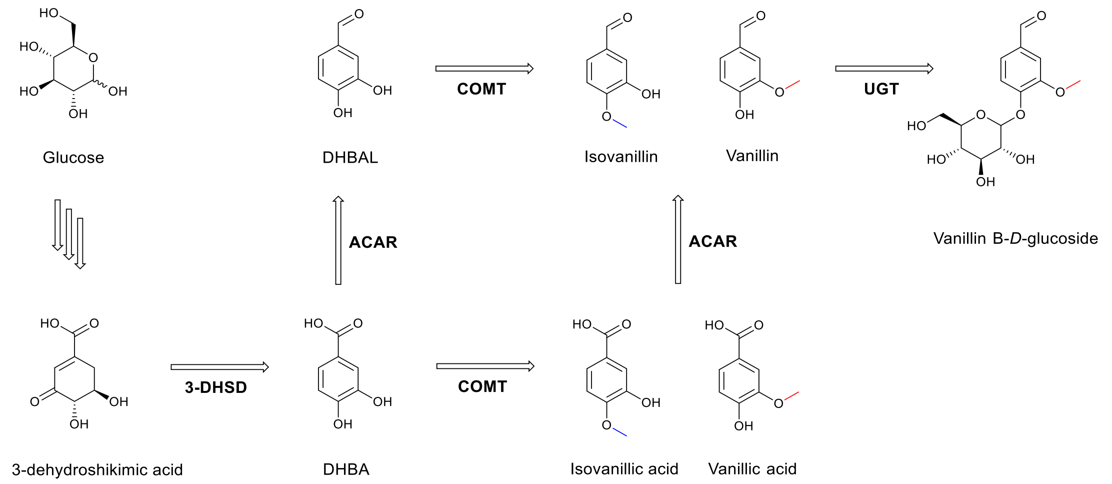

Figure S1. Engineered biosynthetic pathway to vanillin from glucose in fission yeast.[3] 3DHSD: 3-dehydroshikimate dehydratase; ACAR: aromatic carboxylic acid reductase; UGT: UDP-glycosyltransferase.

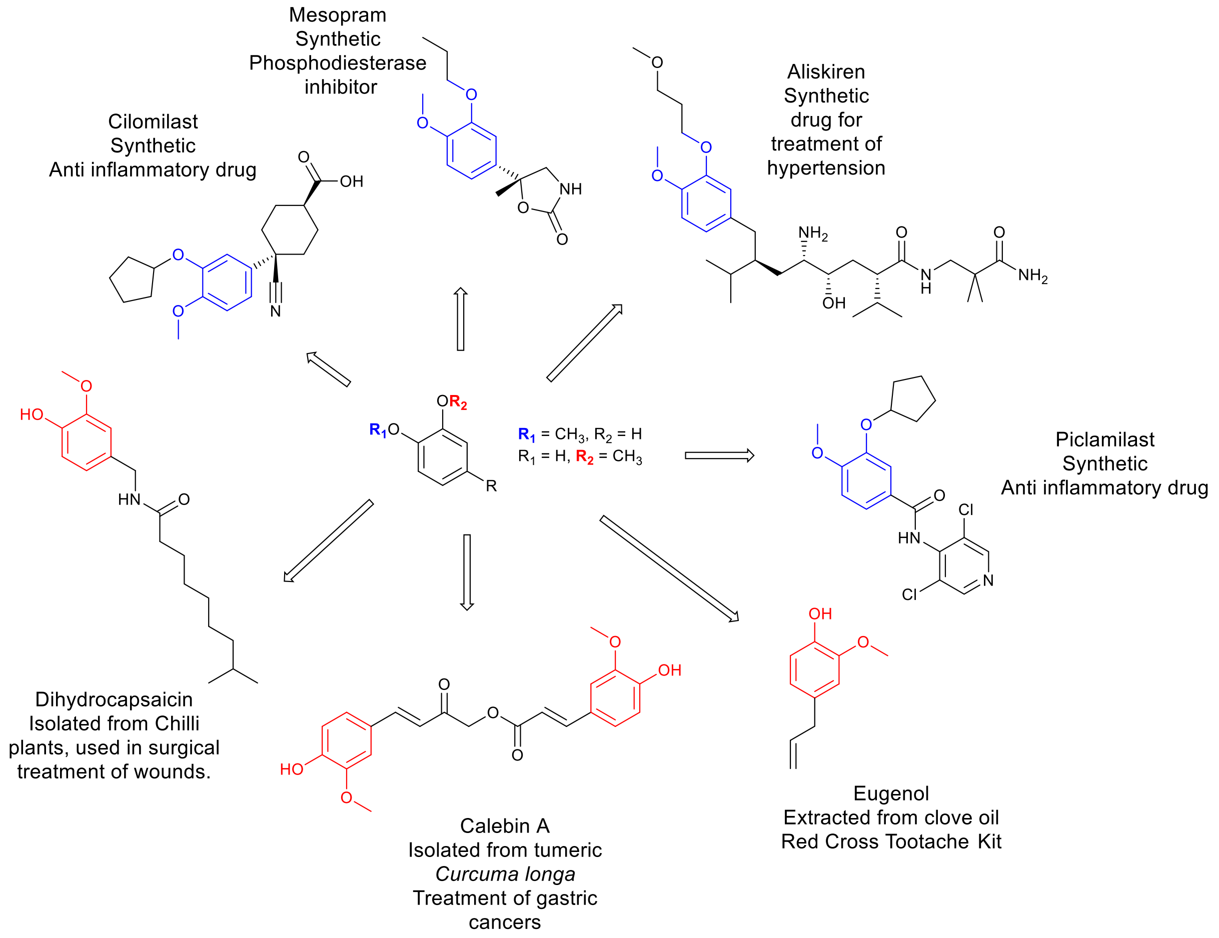

Figure S2. Methylated catechols as potential building blocks in APIs.[S7–S12]

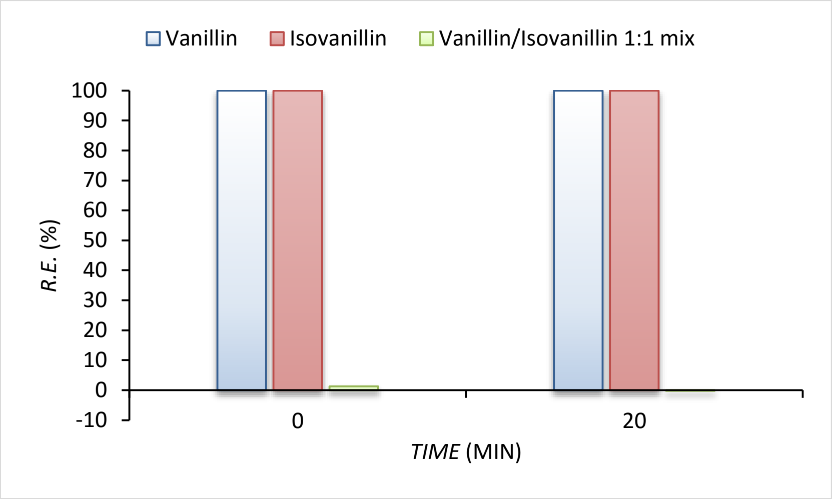

Figure S3. The COMT-catalysed reaction does not appear to be reversible. Incubation of WT COMT with vanillin **2a** (blue bars) or isovanillin **3a** (red bars) with SAH did not result in the formation of the other regioisomer after 20 min. Incubation of COMT with equimolar **2a** and **3a** (green bars) did not show a change in the ratios of the two regioisomers.

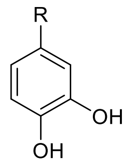

1a R = CHO
1b R = COOH
1c R = CH_2COOH
1d R = NO_2

1a-d

Table S1. Percentage conversions and regiomeric excesses of COMT mutants with substrates **1a-d**. **1a** and **1b** are key intermediates in the biosynthesis of vanillin from glucose, **1c** possesses an ionisable R-group that can be influenced by the active site mutations introduced, whereas **1d** was selected due to its potential as a compound for development of colormetric screens for methyltransferase activity.

	**DHBAL 1a**		**DHBA 1b**		**DHPA 1c**		**NOC 1d**	
	% conv.	% r.e.	% conv.	% r.e.	% conv.	% r.e.	% conv.	% r.e.
**WT**	100	54	94	58	72	82	83	72
**W38A**	59	91	5.4	54	1.0	41	48	91
**W38D**	51	93	2.8	70	0.4	57	41	93
**W38F**	93	64	35	55	11	36	82	73
**W38H**	82	80	35	5.4	13	-8.2	68	82
**W38K**	74	79	36	-16	13	-17	51	81
**W38L**	69	87	17	36	4.3	28	63	87
**W38R**	78	78	47	-39	20	-40	57	81
**W38Y**	85	86	27	20	7.8	-34	80	90
**K144A**	36	19	4.1	61	1.0	83	48	-4.9
**K144A/V173Y**	39	-0.6	4.1	54	1.3	86	47	-40
**V173F**	98	17	97	59	86	83	80	40
**V173W**	89	69	90	66	75	88	62	69
**V173Y**	94	26	96	50	83	78	63	41
**E199D**	85	86	30	67	10	77	72	86
**E199L**	78	85	14	63	1.2	67	59	85
**Y200A**	84	88	26	65	8.6	88	72	85
**Y200D**	89	91	12	61	1.6	83	92	93
**Y200F**	91	83	33	71	10	89	77	83
**Y200L**	87	90	23	76	4.7	91	73	90
**Y200R**	90	83	35	6.9	13	61	69	83
**Y200W**	68	90	16	66	4.4	87	53	90

Conversions were calculated as the percentage of substrate converted to *meta*- and *para*-products combined. Regioisomer excesses (r.e.s) were calculated as the percentage excess of the major regioisomer over the minor regioisomer. Positive r.e.s denote a regiomeric excess of the *meta*-isomer, whereas negative r.e.s denote excess of the *para*-isomer.

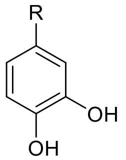

1a R = CHO
1b R = COOH
1c R = CH_2COOH
1d R = NO_2

1a-d

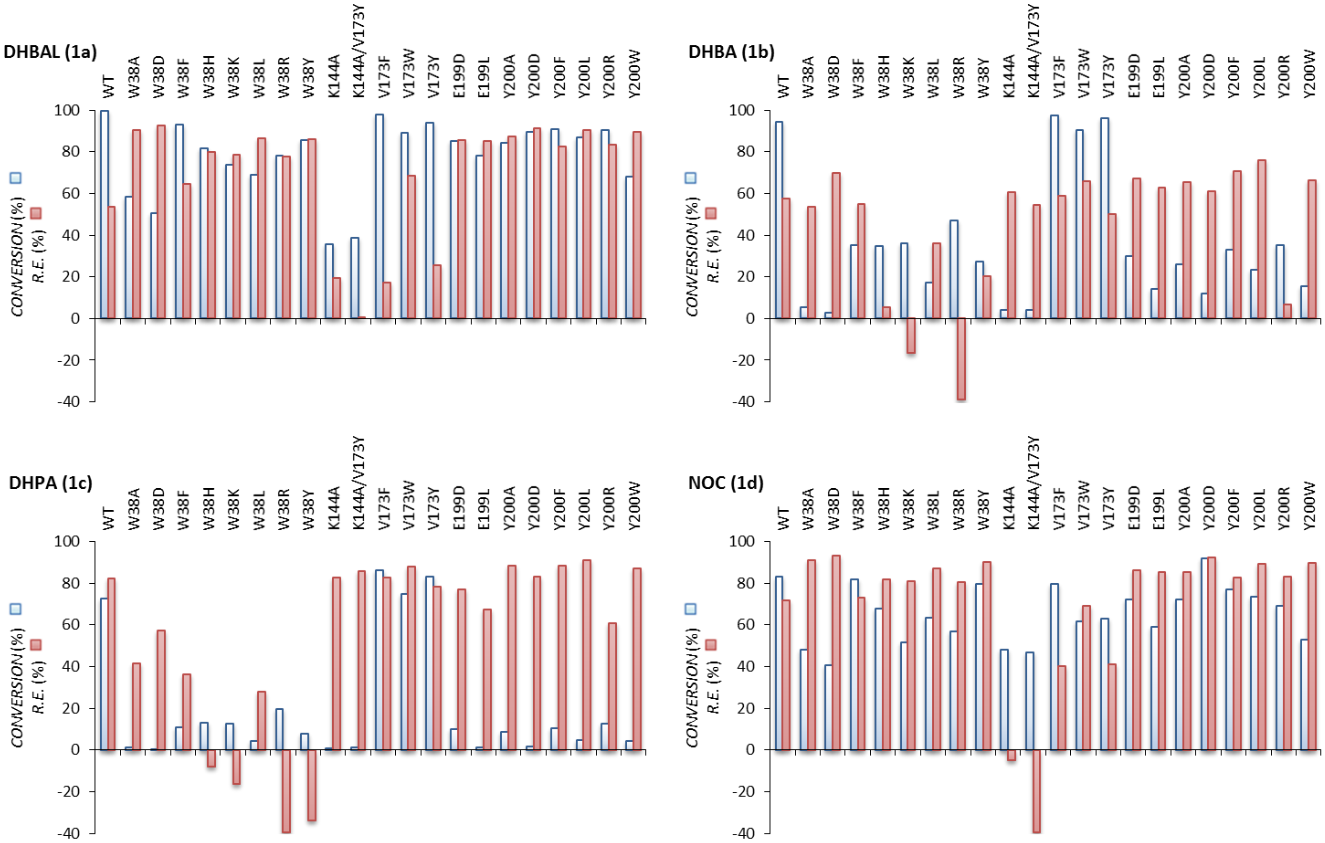

Figure S4. Comparison of activity and regioselectivity between substrates **1a-d** for all COMT mutants. Conversions are shown as blue bars, r.e.s are shown as red bars. Conversions were calculated as the percentage of substrate converted to *meta*- and *para*-products combined. Positive r.e.s denote a regioisomer excess of the *meta*-regioisomer, whereas negative r.e.s denote excess of the *para*-regioisomer.

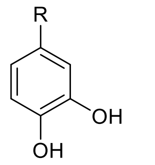

1a R = CHO
1b R = COOH
1c R = CH_2COOH
1d R = NO_2

1a-d

Table S2. Michaelis-Menten kinetics of WT COMT, WT COMT monomeric and dimeric forms, and mutants. Kinetic parameters were determined for those mutants that showed most significant shifts in regioselectivity. Mutations W38D and W38R perturbed the binding of substrates DHBAL **1a** and NOC **1d** to a greater extent, leading to higher K_m values. This is consistent with the structural studies which suggest that W38 could π-stack with the catechol ring and thereby contribute towards substrate binding.[5a, S13, S14] The K_m values for Y200L with **1a** and **1d** are similar to those of the wild-type enzyme; Y200 is more distal to the catechol ring and is thus less likely to make contact with and affect the substrate binding affinity. The K_m values were significantly higher for all mutants with DHBA **1b**, indicating that the binding of this substrate is more sensitive to changes in the active site. The k_{cat} values for W38D, W38R and Y200L were similar to that of the wild-type, whilst K144 mutants generally exhibited lower catalytic rates. Surprisingly, given the postulated role of K144 as the catalytic general base[5a, S14, S15] significant enzyme activity was still retained with K144 mutants (Fig. 2). Indeed with **1d** as a substrate, K144A/V173Y exhibited similar turnover number to the WT COMT, albeit with lower efficiency. This suggests that whilst K144 may participate in deprotonating the catechol hydroxyl group, it is not essential. Presumably the coordination of the hydroxyl groups with the Mg^{2+} cation, and the presence of the more electron-withdrawing nitro group of **1d** can serve to lower the pK_a of the substrate hydroxyl group sufficiently to allow a water molecule to abstract the proton. To explore this phenomenon further, the effect of pH on enzyme activity was explored with the wild-type and K144A mutant and the substrate **1a** (Fig. S5).

Substrate	Enzyme	K_m (μM)	k_{cat} (min^{-1})	k_{cat}/K_m (e−02 min^{-1} μM^{-1})
DHBAL **1a**	WT	3.4 ± 0.32	4.0 ± 0.083	120 ± 11
	WT monomer	1.3 ± 0.18	2.3 ± 0.050	180 ± 25
	WT dimer	3.7 ± 0.28	3.2* ± 0.055	87 ± 6.9
	Y200L	5.7 ± 0.29	5.5 ± 0.073	97 ± 5.0
	K144A	110 ± 12	1.1 ± 0.31	1.1 ± 0.12
	K144A/V173Y	55 ± 3.1	1.1 ± 0.029	2.0 ± 0.13
	W38D	140 ± 19	4.3 ± 0.15	3.1 ± 0.43
	W38R	35 ± 2.3	5.0 ± 0.068	14 ± 0.97
DHBA **1b**	WT	30 ± 1.9	8.5 ± 0.15	29 ± 1.8
	Y200L	610 ± 45	3.5 ± 0.078	0.57 ± 0.044
	K144A/V173Y	490 ± 13	0.57 ± 0.0042	0.12 ± 0.0032
	W38R	280 ± 20	6.6 ± 0.11	2.4 ± 0.18
NOC **1d**	WT	1.0 ± 0.15	1.9 ± 0.054	180 ± 27
	Y200L	1.5 ± 0.089	3.0 ± 0.038	200 ± 12
	K144A/V173Y	26 ± 1.2	2.6 ± 0.028	9.8 ± 0.47
	W38D	29 ± 2.0	1.7 ± 0.026	5.6 ± 0.39
	W38R	6.4 ± 0.51	2.1 ± 0.035	32 ± 2.7

Note that k_{cat}/K_m values are given as *x* e−02. Kinetics data for DHBAL **1a** were obtained with both K144A and K144A/V173Y. The double mutant was subsequently assayed with substrates DHBA **1b** and NOC **1d**, in order to study the effects of K144 mutations on catalytic efficiency. We were unable to obtain data for K144A with **1b** due to poor substrate binding; whereas with K144A/V173Y, presumably additional catechol ring π-interactions with V173Y lead to enhanced binding affinity compared with the K144A mutant alone.*k_{cat} of dimeric form containing two active sites.

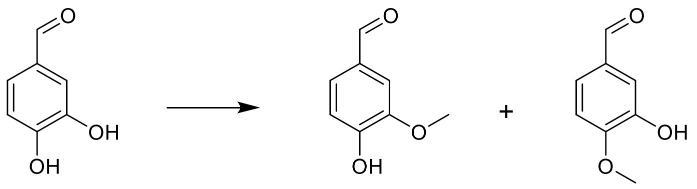

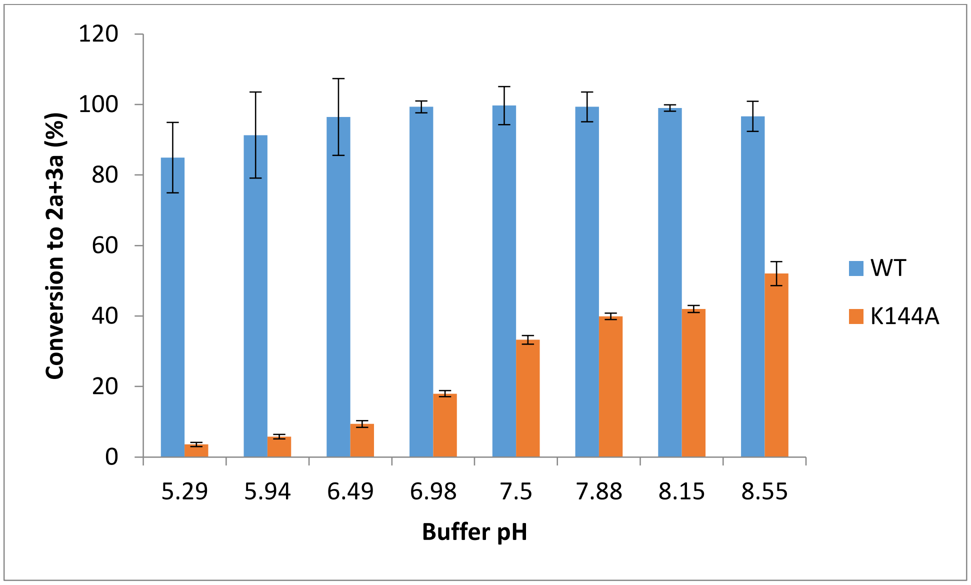

Figure S5. pH profile of WT and K144A COMT activity with substrate DHBAL **1a**. The WT and K144A enzymes were assayed with substrate **1a** over a pH range of 5.29 to 8.55. The conversion of **1a** to *meta*- and *para*-methylated products combined for each enzyme at each pH was plotted to give the pH dependent activity profile. Over a pH range of 5.3-8.6, little change in activity occurred with the wild-type enzyme, but a marked increase in activity was observed with increasing pH for the K144A mutant. Previous literature has suggested that K144 acts as a catalytic base to deprotonate the hydroxyl group closest to the AdoMet sulphonium centre,[S14-S16] with the aid of Mg^{2+} coordination lowering the hydroxyl pK_as.[5a] We suggest that in the absence of K144, increasing solvent pH results in greater ionization of the catechol hydroxyl and faster methylation. With the wild-type enzyme, the presence of K144 as a general base results in substrate deprotonation that is independent of the solvent pH.

Table S3. Refinement statistics and X-ray diffraction data for COMT: Y200L mutant with DNC, and wild-type dimer with 3,5-dinitrocatechol (DNC).

	Y200L + DNC	WT COMT dimer + DNC
**Wavelength**	0.92	0.92
**Resolution range**	48.71 - 1.63 (1.688 - 1.63)	38.9 - 1.63 (1.688 - 1.63)
**Space group**	P 21 21 21	P 32 2 1
**Unit cell**	61.7 79.37 109.66 90 90 90	50.72 50.72 167.55 90 90 120
**Total reflections**	498019 (51143)	195761 (19819)
**Unique reflections**	66577 (6546)	31410 (3088)
**Multiplicity**	7.5 (7.8)	6.2 (6.4)
**Completeness (%)**	98(98)	93 (97)
**Mean I/sigma(I)**	10.63 (2.98)	16.69 (2.20)
**Wilson B-factor**	13.08	21.55
**R-merge**	0.139 (0.756)	0.05378 (0.7007)
**R-meas**	0.149 (0.809)	0.0582 (0.7585)
**CC1/2**	0.997 (0.821)	0.999 (0.813)
**CC***	0.999 (0.95)	1 (0.947)
**Reflections used in refinement**	66577 (6545)	29973 (2613)
**Reflections used for R-free**	3377 (367)	1504 (140)
**R-work**	0.159 (0.206)	0.155 (0.257)
**R-free**	0.178 (0.237)	0.188 (0.285)
**CC(work)**	0.967 (0.885)	0.976 (0.916)
**CC(free)**	0.961 (0.850)	0.970 (0.815)
**Number of non-hydrogen atoms**	4170	1974
**Macromolecules**	3404	1706
**Ligands**	31	15
**Protein residues**	426	213
**RMS(bonds)**	0.007	0.014
**RMS(angles)**	1.17	1.55
**Ramachandran favored (%)**	97	97
**Ramachandran allowed (%)**	3.1	2.8
**Ramachandran outliers (%)**	0	0
**Rotamer outliers (%)**	0.82	1.1
**Clashscore**	4.22	0.88
**Average B-factor**	16.67	27.34
**Macromolecules**	14.31	25.4
**Ligands**	15.45	25.48
**Solvent**	27.69	40.52

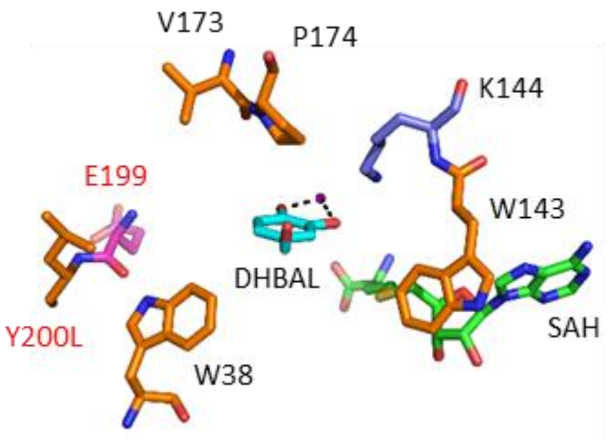

Figure S6. Model of the active site of Y200L with SAH and active substrate DHBAL **1a** based on a preliminary crystal structure. The binding modes of **1a** and SAH are consistent with those observed in the structures of Y200L and WT dimer with DNC and AdoMet. The loss of E199 interaction with the aldehyde of **1a** leads to preferential binding in the orientation for *meta*-methylation i.e. the aldehyde is positioned at the solvent interface.

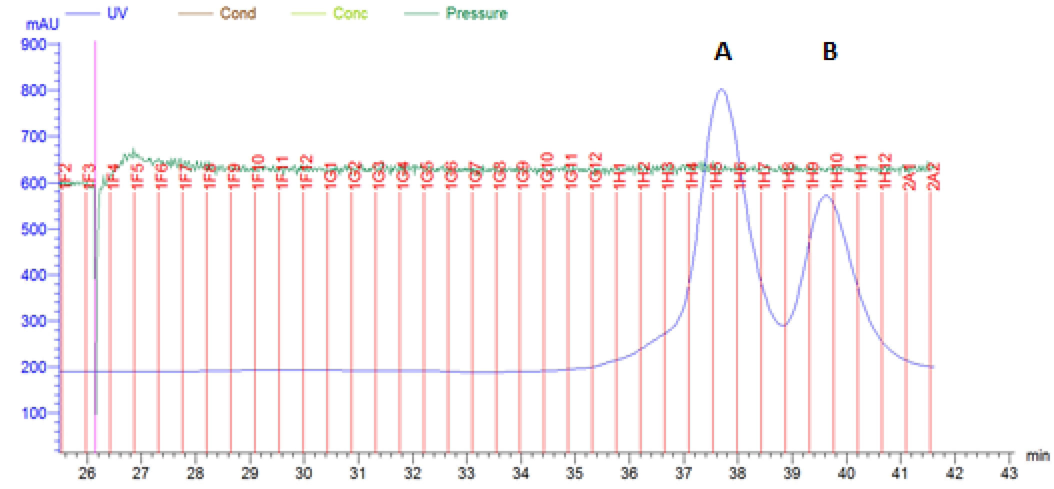

Figure S7. FPLC chromatogram showing dimeric (A) and monomeric (B) forms of COMT.

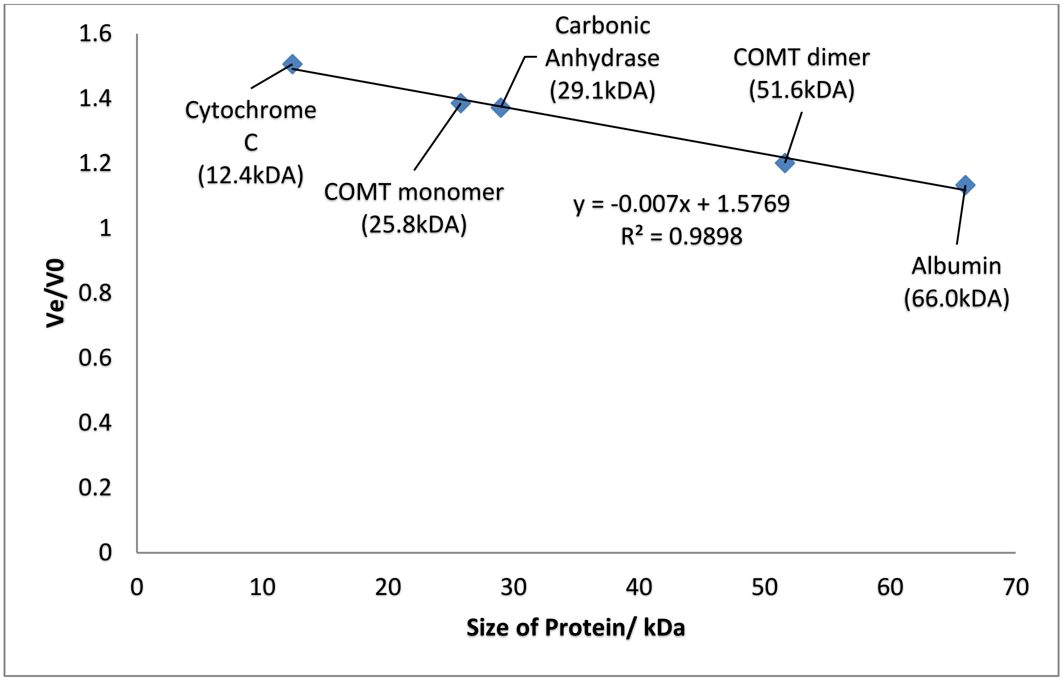

Figure S8. Gel filtration calibration with protein standards. The calibration curve was plotted with a series of proteins with known molecular weights, and used to verify the molecular weights of COMT monomeric and dimeric forms as predicted by gel filtration FPLC.

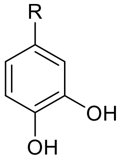

1a R = CHO
1b R = COOH
1c R = CH_2COOH
1d R = NO_2

1a-d

Table S4. Activity and regioselectivity of wild-type COMT monomer and dimer.

Substrate	Monomer activity %	Monomer r.e. %	Dimer activity %	Dimer r.e. %
1a	49	+31	31	+84
1b	64	+48	21	+59
1d	49	+33	50	+87

The monomeric and dimeric forms of WT COMT were separated by gel filtration FPLC and assayed with substrates **1a**, **1b** and **1d** at 37 °C, 800 rpm shaking, 20 min. Conversions and r.e.s were calculated as described previously. Kinetic parameters were also determined for monomeric and dimeric COMT with **1a** (Table S2). The monomeric form showed a higher catalytic rate per active site (2.3 ± 0.050 min^{-1}) compared with the dimer (1.6 ± 0.030 min^{-1}), and a lower K_m (1.3 ± 0.18 μM monomer, 3.7 ± 0.28 μM dimer), as reflected in the activity assays shown above. Thus, monomeric COMT appears to bind more tightly and with greater reaction turnovers, yet exhibits considerably lower regioselectivity than the dimer.

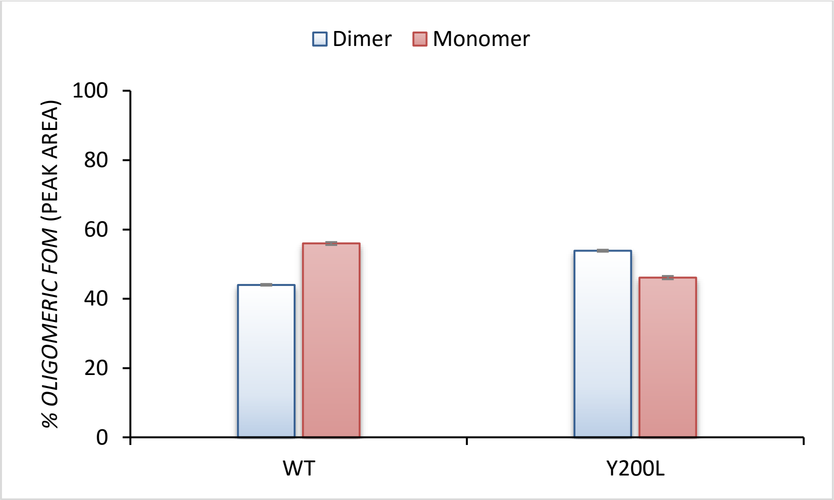

Figure S9. Oligomeric forms of WT and Y200L in solution. The ratio of dimer to monomer by peak area for WT COMT was determined to be 0.8:1, whereas for Y200L the monomer:dimer ratio was 1.1:1.

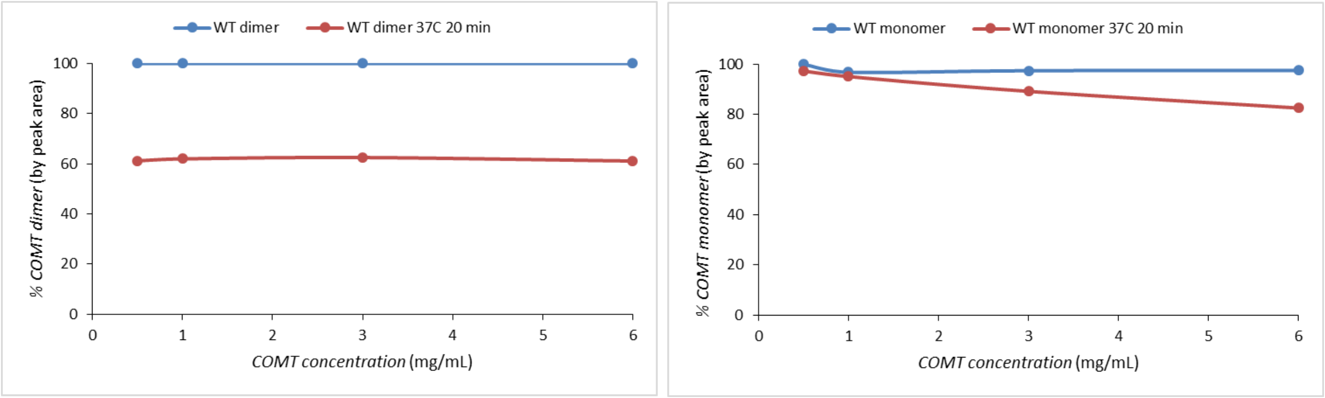

Figure S10. Stability of COMT WT dimer and monomer under dilution and incubation under assay conditions. The dimeric and monomeric forms were isolated and diluted from the gel filtration FPLC purification concentration (~6 mg/mL 232 µM) to an approximate assay concentration of 0.5 mg/mL (19 µM). Dilution does not appear to cause any change in oligomeric state, with both dimer and monomer remaining at 100% (blue lines). Incubation of the same samples for 20 min under assay conditions (37 °C, 800 rpm agitation) results in a decrease in the amount of the original oligomeric state (red lines), as some of the other oligomeric state begins to be formed.

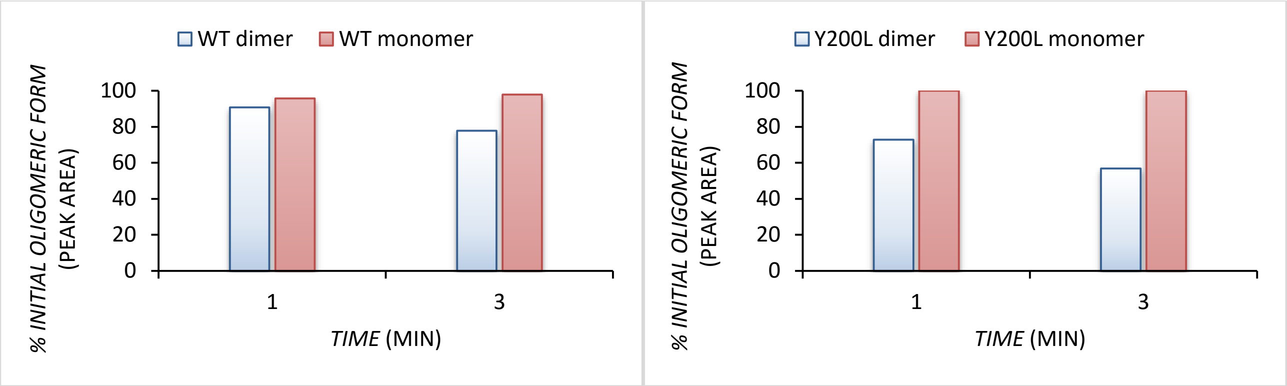

Figure S11. Stability of WT and Y200L COMT oligomeric forms over time. The monomeric and dimeric forms were separated, diluted to approximately 15 µM and incubated at 37 °C with 800 rpm agitation for one and three minutes before analysing by gel filtration FPLC. Both WT and Y200L monomer and dimer remained mostly stable over a shorter time period (one minute) and in their initial oligomeric state.

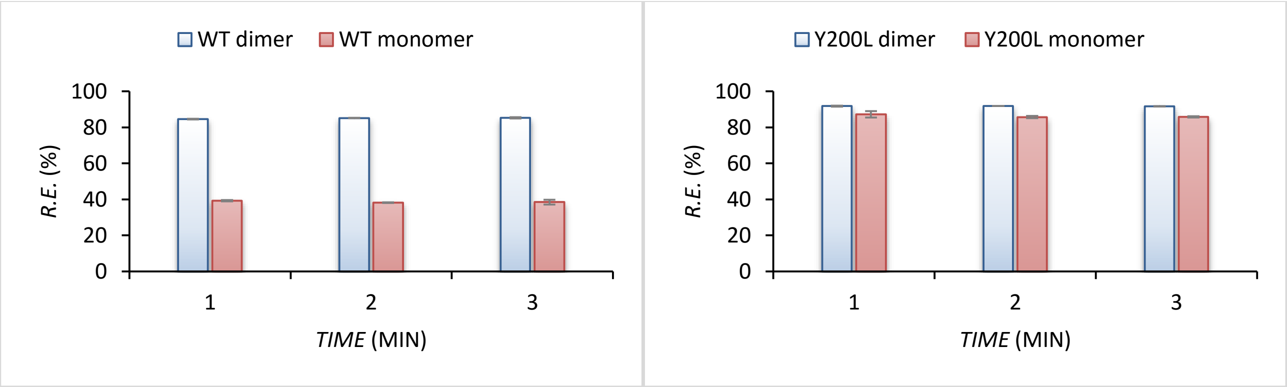

Figure S12. Regioselectivity of monomeric and dimeric COMT over time. The monomeric and dimeric forms of WT and Y200L COMT were assayed with DHBAL **1a** for one, two and three minutes (15 µM enzyme, 0.5 mM **1a**). The WT monomer showed low regioselectivity (+39% r.e.) whereas the dimer showed a high r.e. of +85%. In contrast, with Y200L both monomeric and dimeric forms possessed high r.e.s of +86 and +92% respectively.

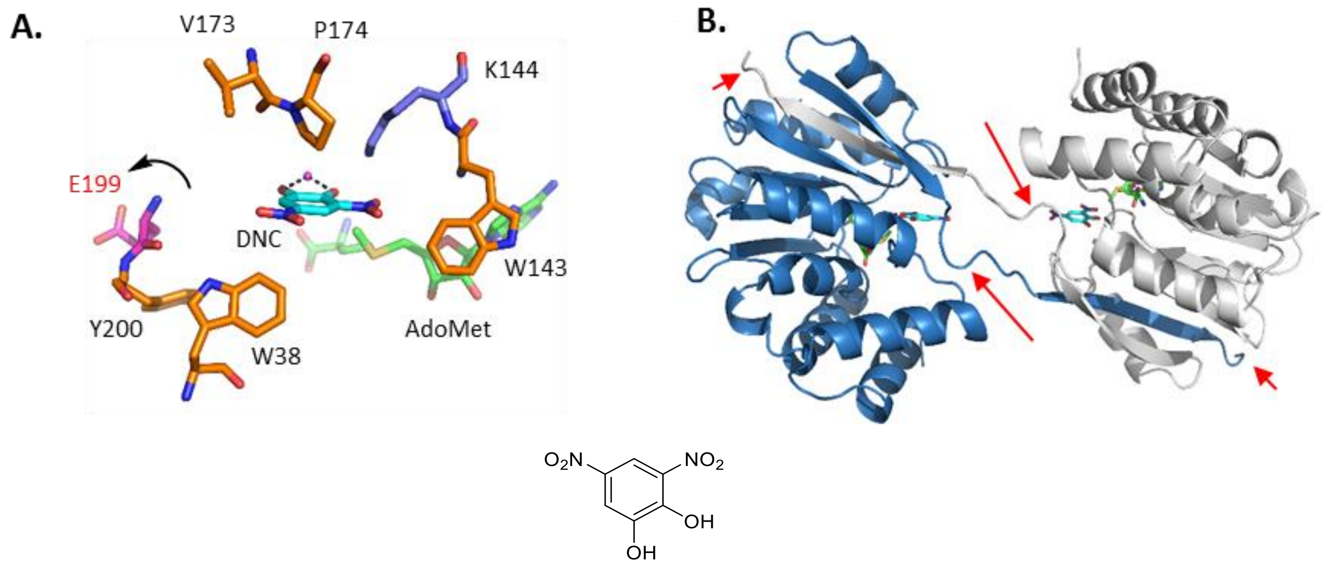

3,5-dinitrocatechol (DNC)

Figure S13. X-ray crystal structure of dimeric WT COMT with DNC bound. **A:** active site, with the E199 residue flipped out in a similar fashion as the Y200L mutant, despite the presence of the Y200 residue. **B:** quaternary structure, showing domain swapping of β7 strands at the C-termini of both subunits (indicated with short arrows), and the E199-Y200 region pulled out of the active site (long arrows). Given that the wild-type dimeric form shows the E199 residue flipping out of the active site in a similar manner to that observed with the Y200L mutant, it is unsurprising that the higher K_m for Y200L with DHBAL **1a** is also reflected in wild-type dimeric COMT (Table S2), and that the r.e.s of dimeric COMT with **1a** and **1d** are similar to that of Y200L (Table S1, Fig. S11).

The regioselectivity assay data for the WT and mutant COMT enzymes described in Fig. S4 and Table S1 were obtained using protein solutions prepared from Ni-NTA purifications. Further purification of WT COMT by gel filtration FPLC revealed that in solution, COMT exists as a mixture of monomeric and dimeric forms (Fig. S7). Separation of the WT monomer and dimer and determining the regioselectivity of each with substrates **1a**, **1b** and **1d** revealed low r.e.s for the monomeric form but high r.e.s for the dimer (Table S4). In order to investigate whether the Y200L mutation was in itself responsible for loss of the E199-substrate interaction, or whether the mutation was causing the enzyme to preferentially form a *meta*-regioselective dimeric state, the Y200L COMT mutant was also analysed by gel filtration FPLC (Fig. S9). The small difference in the dimer:monomer ratio for Y200L (1.1:1) compared with WT (0.8:1) did not appear to correlate well with the large difference in *meta*:*para* ratios between Y200L (19.5:1) and WT (3.3:1). Separation of the Y200L monomer and dimer by FPLC and incubation under assay conditions (37 °C with 800 rpm agitation) revealed that under a short time duration (one minute), the monomeric and dimeric forms remained mostly stable (Fig. S11). Reactions of Y200L monomer and dimer under the same conditions (one to three minutes duration to ensure stability of the separated monomeric and dimeric forms) with substrate **1a** revealed high *meta*-r.e.s for both forms (Fig. S12) unlike with the WT COMT. Thus, with the Ni-NTA purified COMT assays (Fig. S4, Table S1), the 54% r.e. observed with WT COMT is derived from a mixture of highly regioselective dimer and low regioselectivity monomer. In contrast, Y200L monomer and dimer are both *meta*-regioselective.

The crystal structure of dimeric WT COMT (Fig. S13) reveals movement of E199 out of the active site in a manner similar with that of the Y200L mutant, which was also crystallised as a dimer. However, whereas the WT monomer, with E199 capable of forming an H-bond with the substrate R-group, shows low *meta*-selectivity, the Y200L monomer has a *meta*-selectivity on par with that of both WT and Y200L dimeric forms. Thus, we suggest that with WT COMT the dimerization and domain swapping appear to cause loss of the E199-substrate H-bonding, whereas with Y200L the mutation alone is sufficient, allowing the monomer to be equally regioselective as the dimer.

A.

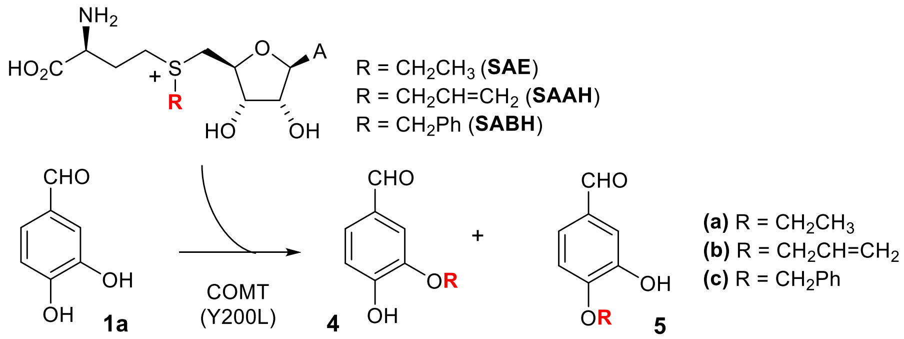

B.

	AdoMet r.e. %	SAE r.e. %	SAAH r.e. %	SABH r.e. %
WT	+54	+43	+45	+33
Y200L	+90	+89	+87	+87

Figure S14. A. Transfer of alkyl groups from AdoMet analogues to DHBAL **1a** using COMT; **B.** Comparison of activity of Y200L COMT with AdoMet analogues, and comparison of regioselectivity of Y200L vs WT COMT with AdoMet analogues.

Previous studies have shown that many AdoMet-dependent methyltransferases,[S17-S23] including COMT,[S24] will accept AdoMet analogues with alternative alkyl sulphonium groups (Fig. S13A). Accordingly, an enzymatic method utilizing a mutant human methionine adenosyl transferase, hMAT2A (I322V)[11b,d], was used to prepare *S*-adenosyl-ethionine (SAE) or *S*-adenosyl-allylhomocysteine (SAAH) from ATP and ethionine or *S*-allyl-homocysteine, respectively[11b-d] whilst *S*-adenosyl-benzylhomocysteine (SABH) was prepared synthetically, using the synthesis of Dalhoff *et al.*[11a] Wild-type COMT was shown to have lower *meta*-regioselectivity with SAE, SAAH and SABH, (r.e.s of +43%, +45% and +33% respectively with **1a**, compared with r.e. +54% with AdoMet). However, with Y200L, the cofactor analogues exhibited considerably improved regioselectivity with SAE, SAAH and SABH (r.e.s of +89%, +87% and +87% respectively, in line with the r.e. for AdoMet of +90% (Fig. S13B)). To demonstrate potential applications of engineered COMT, the reaction of DHBAL **1a** with SAE was optimized with the Y200L mutant to produce ethyl vanillin **4a**, an important flavoring product, in 58% isolated yield. The *in vitro* enzymatic synthesis of ethyl vanillin from **1a**, ATP and ethionine presented here would not compete with current synthetic methods. However, with further research it may be possible to engineer microbial strains producing DHBAL **1a** and DHBA **1b**, with hMAT2A and COMT that could selectively ethylate the catechol substrates upon feeding ethionine, to produce ethyl vanillin in higher titres.

Table S5. Primers used in this study.

Primer	Sequence 5′-3′	bp
W38A F	TACTGCACCCAGAAAGAAGCGGCCATGAAC	30
W38A R	ATCACCAACGTTCATGGCCGCTTCTTTCTG	30
W38D F	TACTGCACCCAGAAAGAAGATGCCATGAAC	30
W38D R	ATCACCAACGTTCATGGCATCTTCTTTCTG	30
W38F F	TACTGCACCCAGAAAGAATTTGCCATGAAC	30
W38F R	ATCACCAACGTTCATGGCAAATTCTTTCTG	30
W38H F	TACTGCACCCAGAAAGAACATGCCATGAAC	30
W38H R	ATCACCAACGTTCATGGCATGTTCTTTCTG	30
W38K F	TACTGCACCCAGAAAGAAAAAGCCATGAAC	30
W38K R	ATCACCAACGTTCATGGCTTTTTCTTTCTG	30
W38L F	TACTGCACCCAGAAAGAACTGGCCATGAAC	30
W38L R	ATCACCAACGTTCATGGCCAGTTCTTTCTG	30
W38R F	TACTGCACCCAGAAAGAACGTGCCATGAAC	30
W38R R	ATCACCAACGTTCATGGCACGTTCTTTCTG	30
W38Y F	TACTGCACCCAGAAAGAATATGCCATGAAC	30
W38Y R	ATCACCAACGTTCATGGCATATTCTTTCTG	30
K144A	GTCTTCCTGGACCACTGGGCAGATCGTTAC	30
V173 BDB F	TGCTGGCAGACAACGTAATCBDBCCGGGTAC	31
V173 VHV R	CGAGAAAGTCCGGAGTACCCGGVHVGATTACG	32
E199D F	CACTACTCCAGCTACCTGGAATACATGAAAG	31
E199D R	GTCGACCACTTTCATGTATTCCAGGTAGC	29
Y200A F	ACTACTCCAGCTACCTGGAAGCGATGAAAGTG	32
Y200A R	ACCGTCGACCACTTTCATCGCTTCCAG	27
Y200D F	ACTACTCCAGCTACCTGGAAGATATGAAAGTG	32
Y200D R	ACCGTCGACCACTTTCATATCTTCCAG	27
Y200F F	ACTACTCCAGCTACCTGGAATTTATGAAAGTG	32
Y200F R	ACCGTCGACCACTTTCATAAATTCCAG	27
Y200L F	ACTACTCCAGCTACCTGGAACTGATGAAAGTG	32
Y200L R	ACCGTCGACCACTTTCATCAGTTCCAG	27
Y200R F	ACTACTCCAGCTACCTGGAACGTATGAAAGTG	32
Y200R R	ACCGTCGACCACTTTCATACGTTCCAG	27
Y200W F	ACTACTCCAGCTACCTGGAATGGATGAAAGTG	32
Y200W R	ACCGTCGACCACTTTCATCCATTCCAG	27

BDB (forward) and VHV (reverse) degenerate codons were used to introduce one of a wide range of amino acid changes to the V173 position.

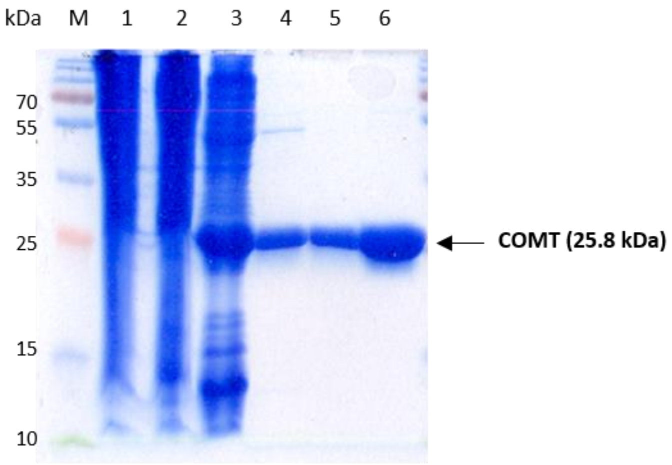

Figure S15. SDS-PAGE of Ni-NTA purified rat S-COMT. Lanes: M) Molecular weight ladder; 1) total cell lysate; 2) soluble cell lysate; 3) flowthrough; 4) 30 mM imidazole wash; 5) 60 mM imidazole wash; 6) 250 mM imidazole elution.

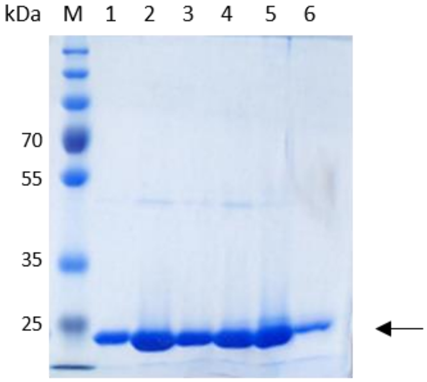

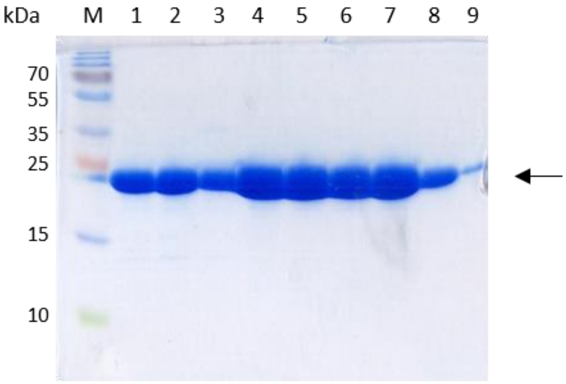

Figure S16. Left: SDS-PAGE of anion exchange purification of rat S-COMT. Lanes: M) Molecular weight ladder; 1-6) fractions containing eluted COMT. Right: SDS-PAGE of gel filtration purification. Lanes: M) Molecular weight ladder; 1-9) fractions containing COMT.

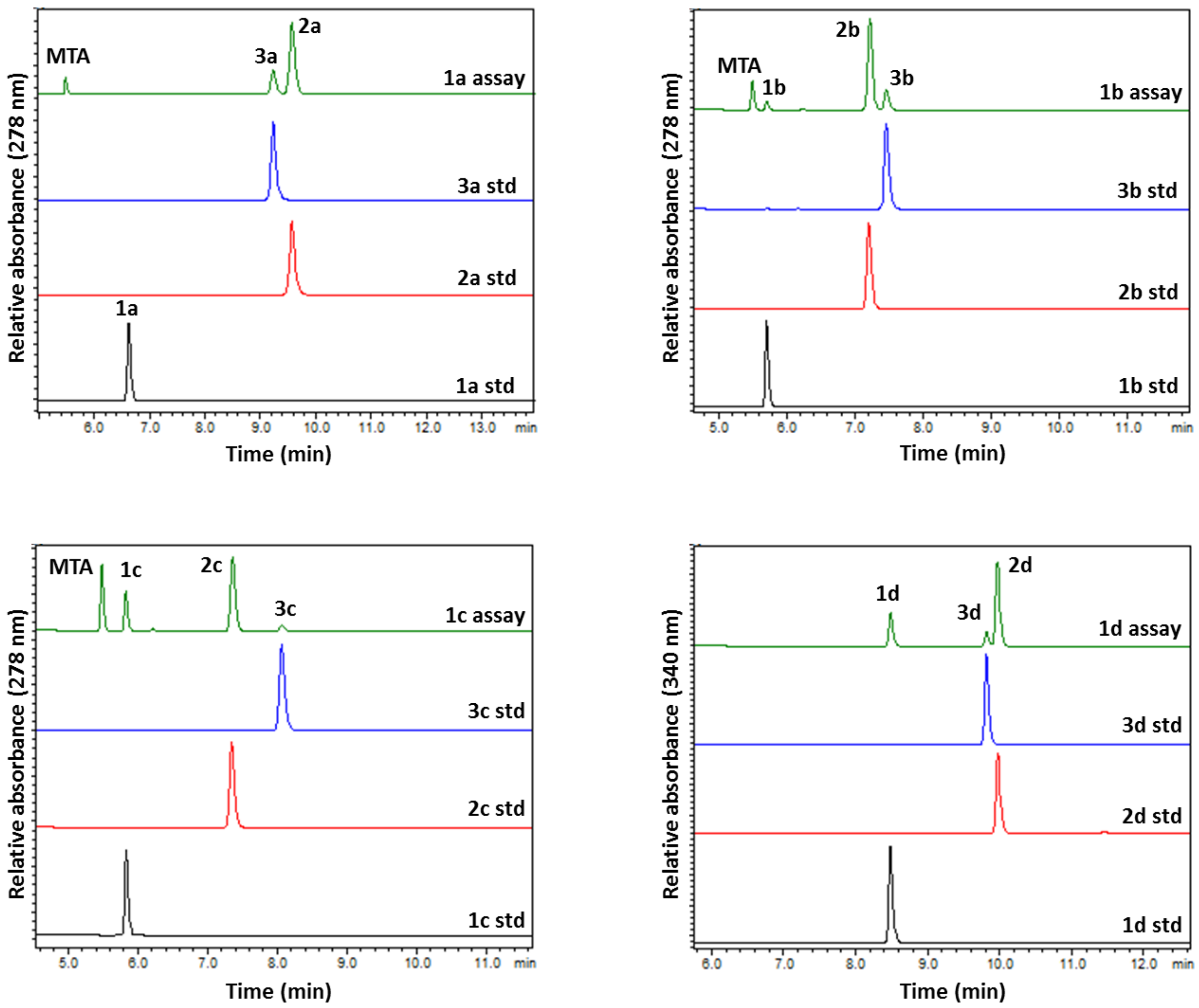

Figure S17. HPLC chromatograms of wild-type COMT assays compared with authentic standards for compounds **1-3**(**a-d**). 5′-*S*-methyl-thioadenosine (MTA) is a degradation product of AdoMet.[S21]

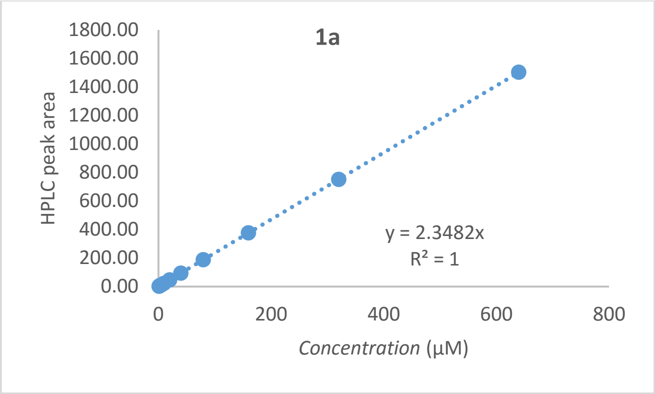

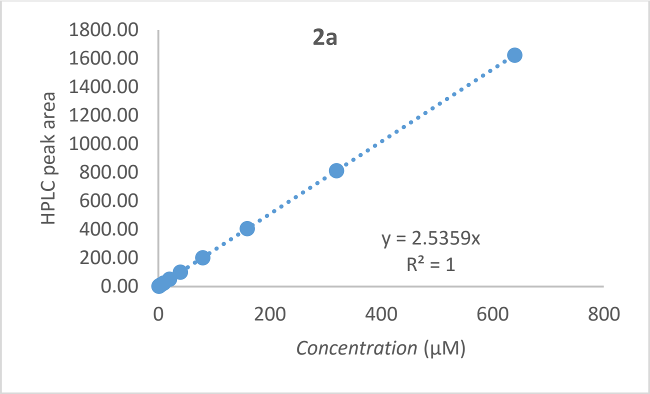

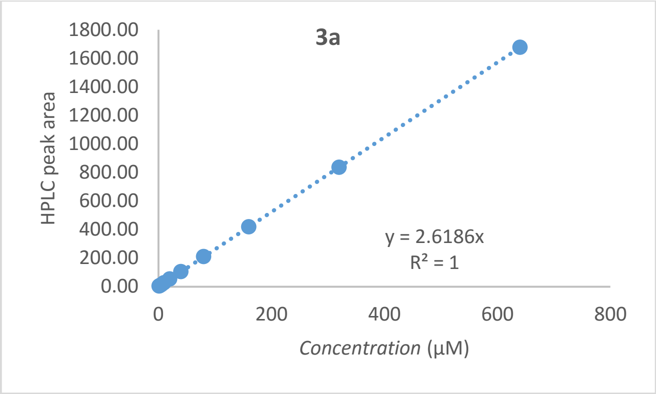

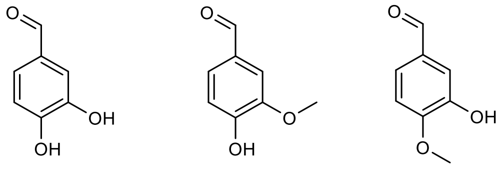

1a **2a** **3a**

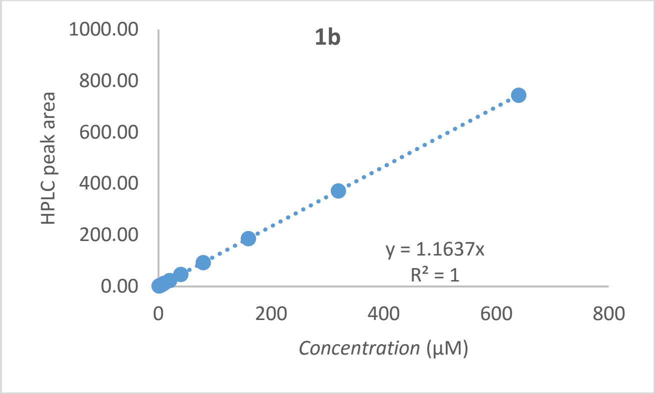

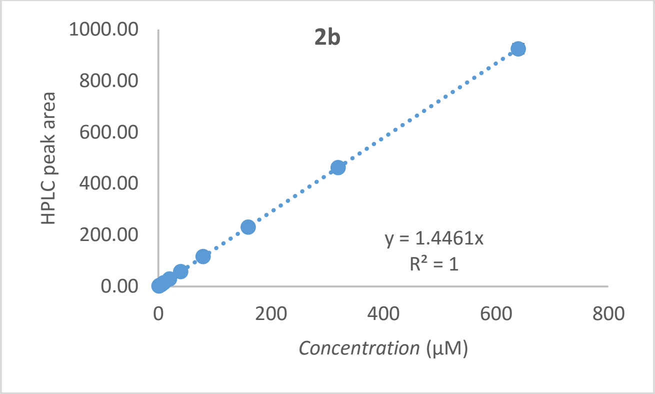

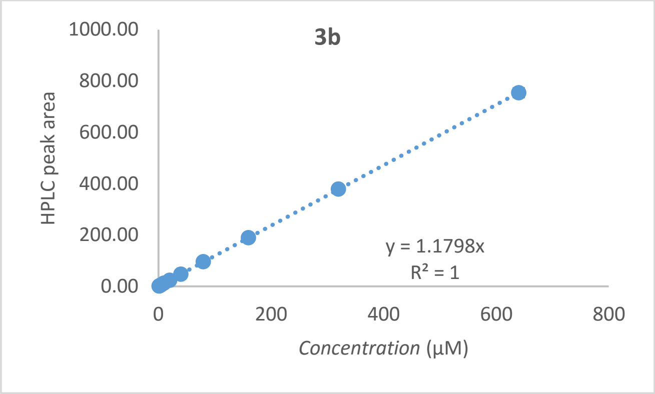

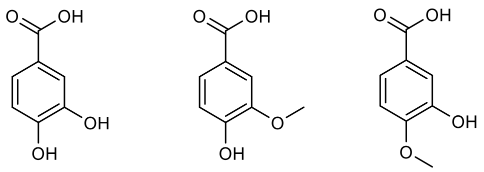

1b **2b** **3b**

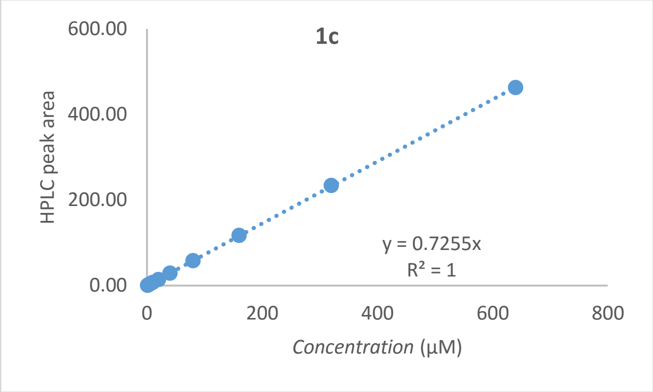

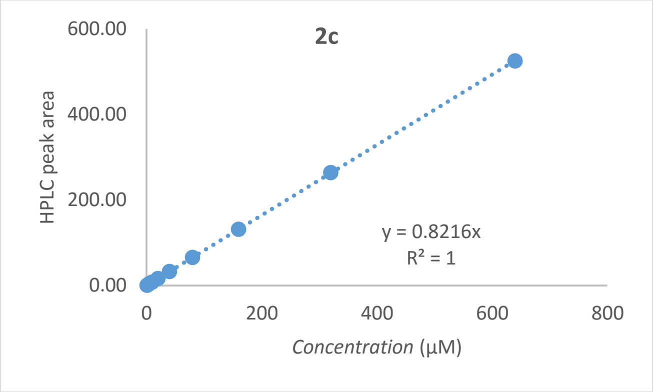

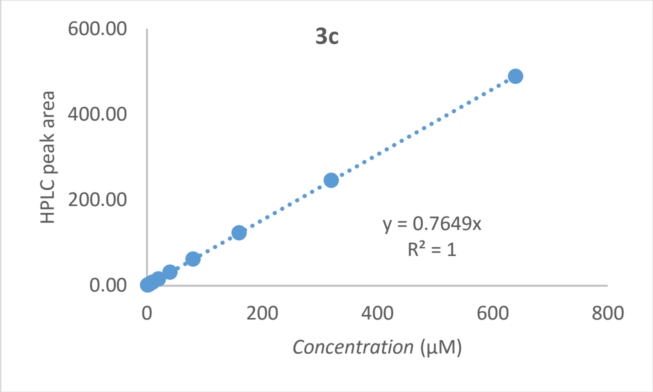

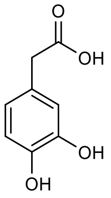

1c

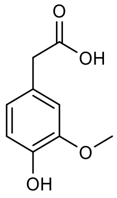

2c

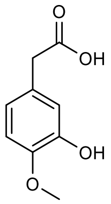

3c

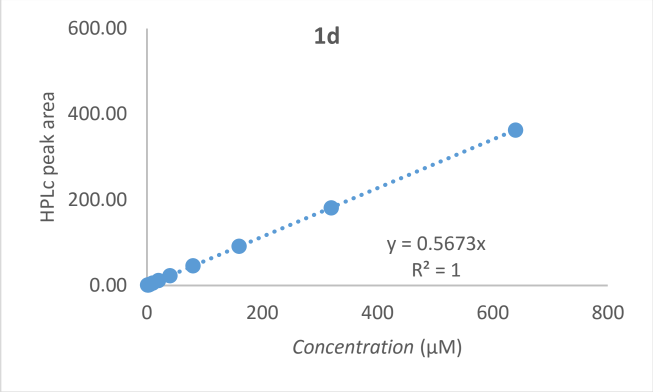

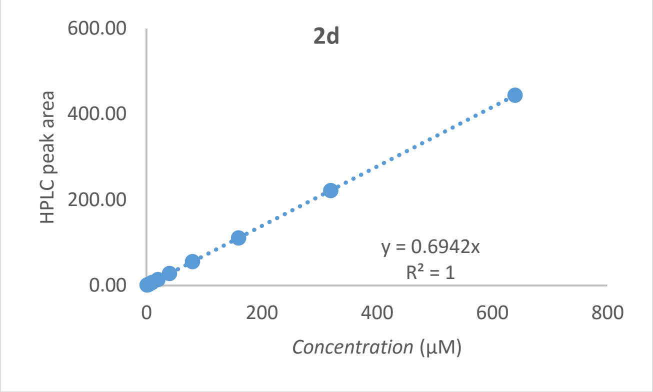

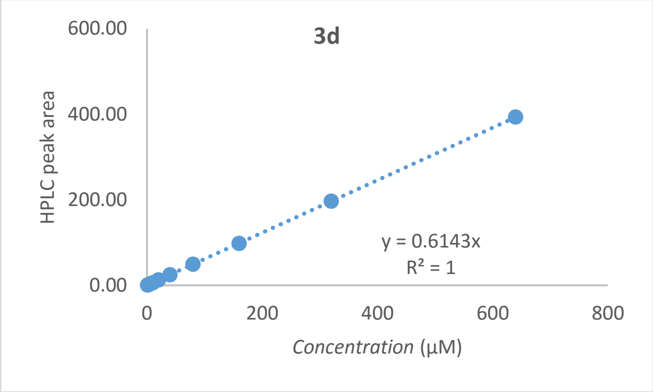

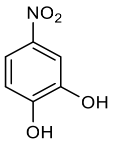

1d

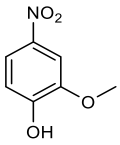

2d

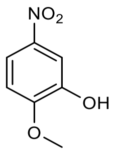

3d

Figure S18. Calibration curves for substrates **1a-d** and products **2-3(a-d)**. Calibrations were used to adjust HPLC peak areas to compensate for differences in extinction coefficients. All calibrations were run in triplicate.

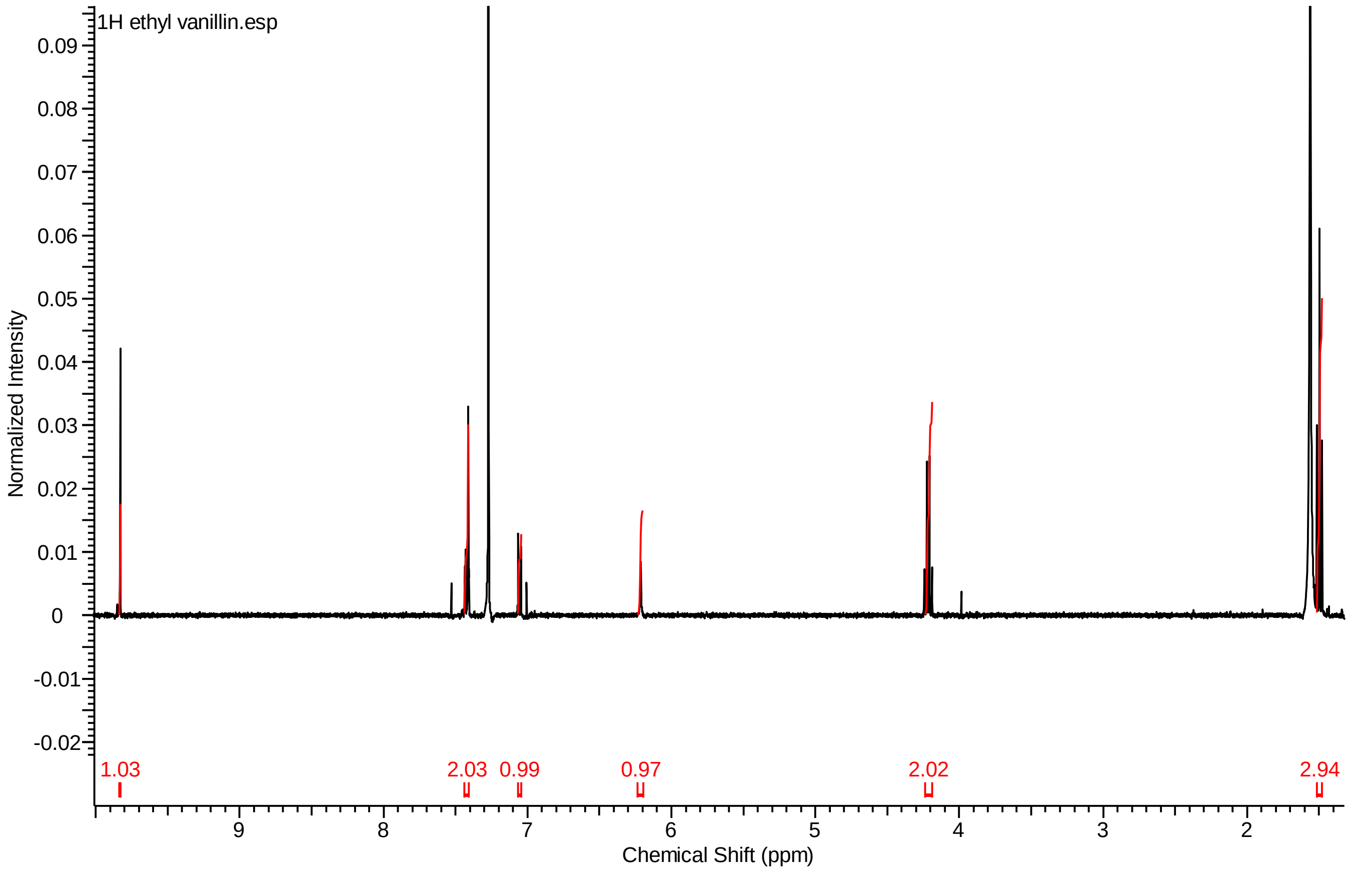

Figure S19. ^{1}H NMR of enzymatically generated ethyl vanillin **4a** in $CDCl_3$.

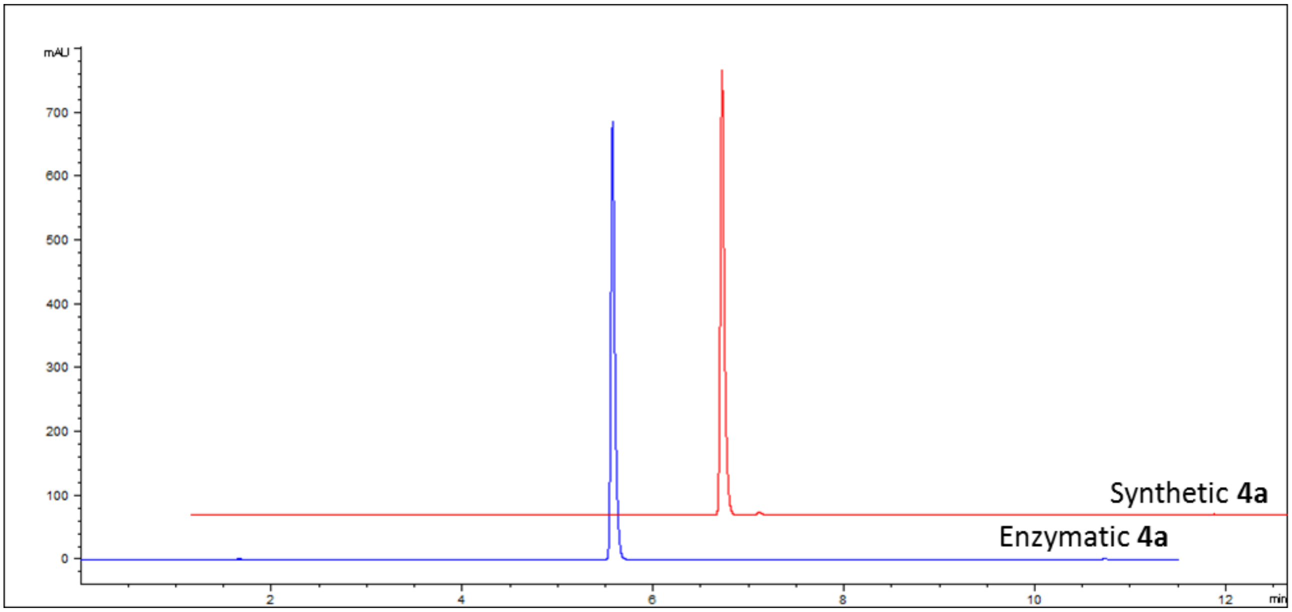

Figure S20. HPLC chromatogram of enzymatic and synthetic ethyl vanillin **4a**, t_R = 5.57 min.

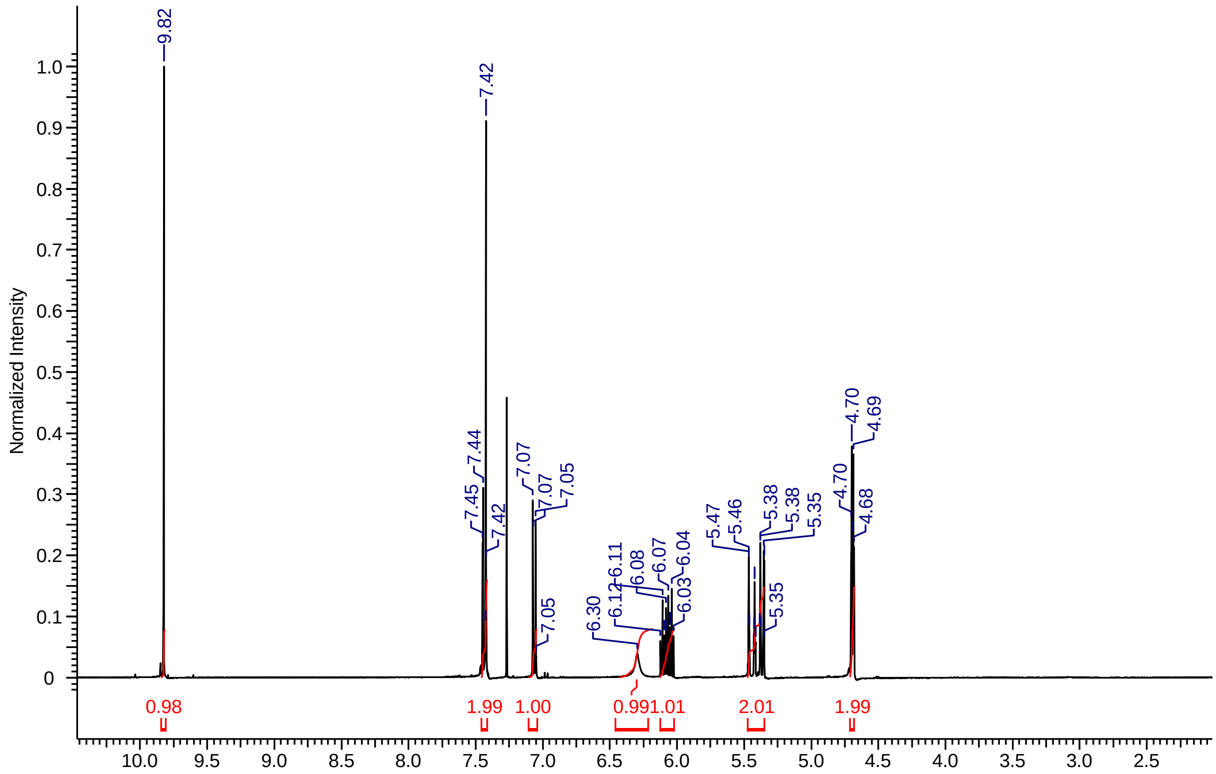

Figure S21. ^{1}H NMR of enzymatically generated allyl vanillin **4b** in $CDCl_3$.

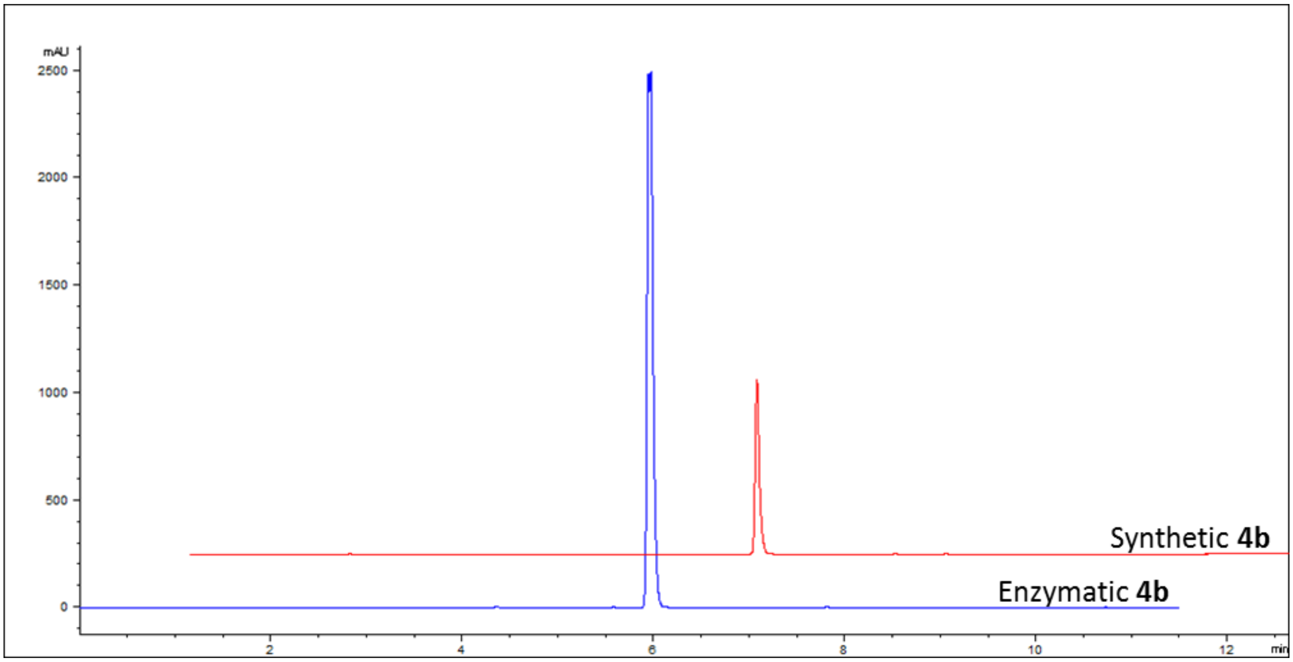

Figure S22. HPLC chromatogram of enzymatic and synthetic allyl vanillin **4b**, t_R = 5.93 min.

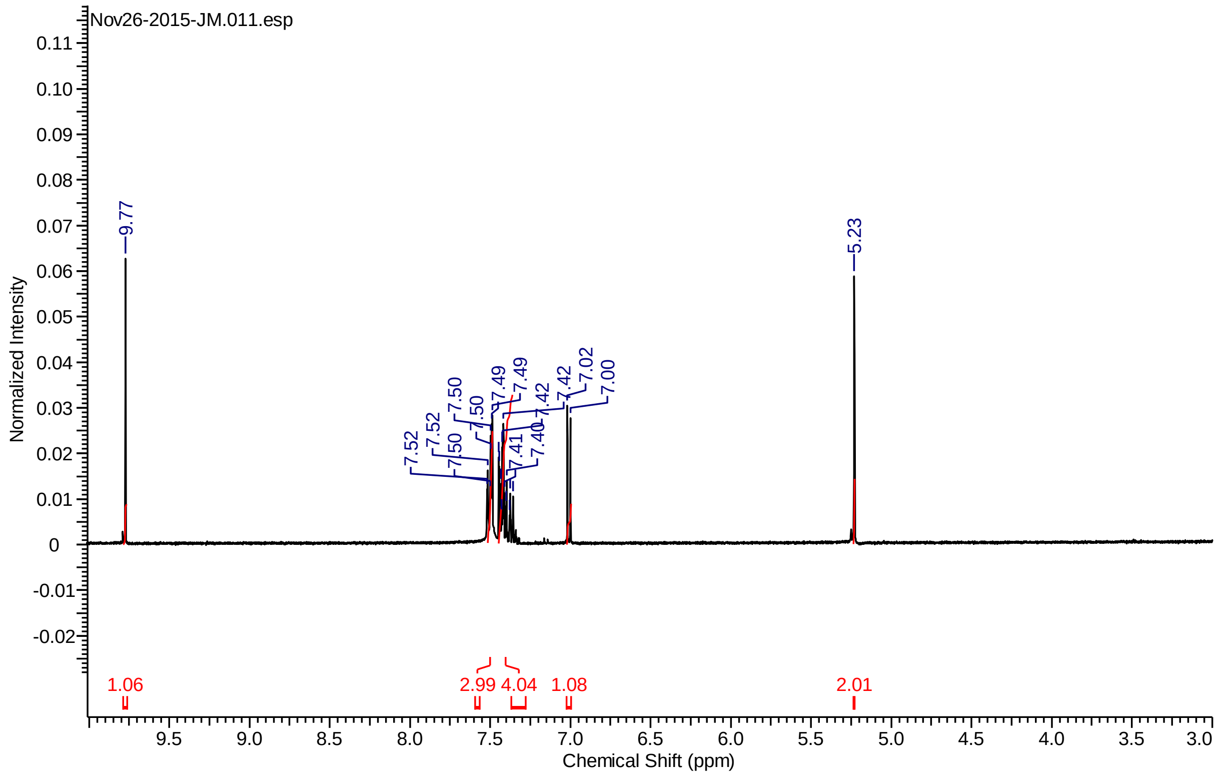

Figure S23. ^{1}H NMR of enzymatically generated benzyl vanillin **4c** in CD_3CN.

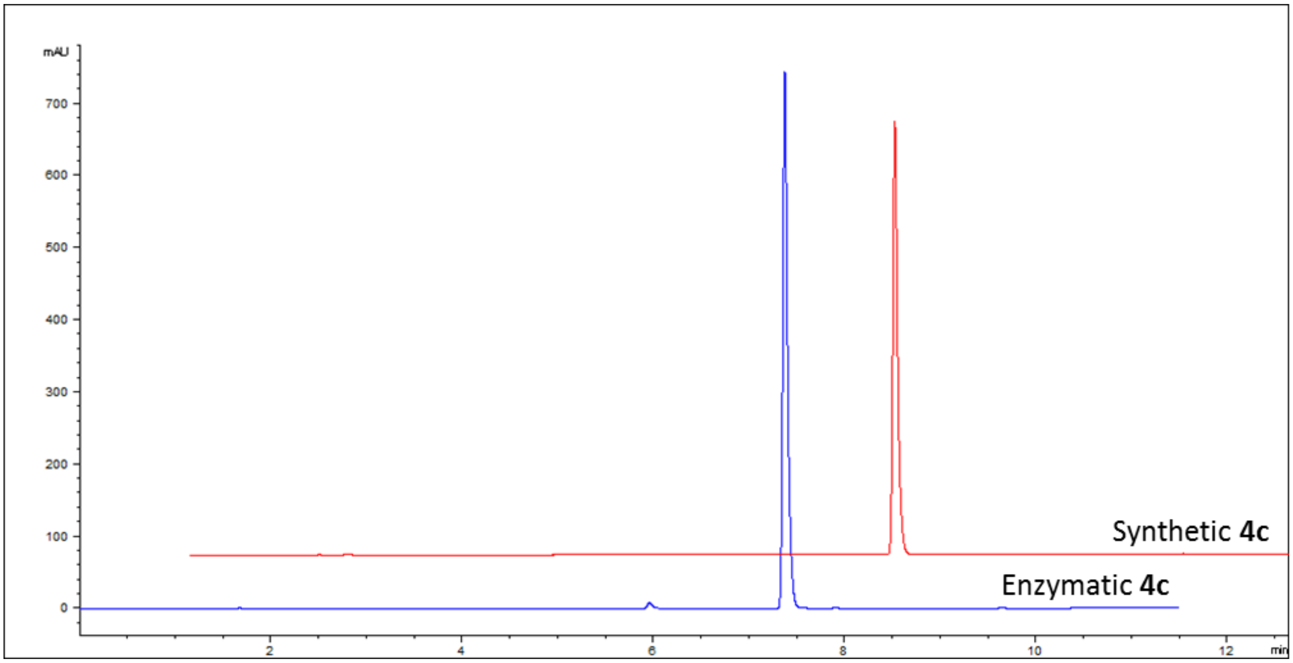

Figure S24. HPLC chromatogram of enzymatic and synthetic benzyl vanillin **4c**, t_R = 7.37 min.

SUPPLEMENTARY REFERENCES

[S1] G. Winter, *J. Appl. Crystallogr.* **2010**, *43*, 186–190.

[S2] A. J. McCoy, R. W. Grosse-Kunstleve, P. D. Adams, M. D. Winn, L. C. Storoni, R. J. Read, *J. Appl. Crystallogr.* **2007**, *40*, 658–674.

[S3] P. Emsley, B. Lohkamp, W. G. Scott, K. Cowtan, *Acta Crystallogr. Sect. D Biol. Crystallogr.* **2010**, *66*, 486–501.

[S4] P. V. Afonine, R. W. Grosse-Kunstleve, N. Echols, J. J. Headd, N. W. Moriarty, M. Mustyakimov, T. C. Terwilliger, A. Urzhumtsev, P. H. Zwart, P. D. Adams, *Acta Crystallogr. Sect. D Biol. Crystallogr.* **2012**, *68*, 352–367.

[S5] V. B. Chen, W. B. Arendall, J. J. Headd, D. a. Keedy, R. M. Immormino, G. J. Kapral, L. W. Murray, J. S. Richardson, D. C. Richardson, *Acta Crystallogr. Sect. D Biol. Crystallogr.* **2010**, *66*, 12–21.

[S6] A. Reitz, M. A. Avery, M. S. Verlander, M. Goodman, *J. Org. Chem.* **1981**, *46*, 4859–4863.

[S7] Y. Li, S. Li, Y. Han, J. Liu, J. Zhang, F. Li, Y. Wang, X. Liu, L. Yao, *Eur. J. Pharmacol.* **2008**, *591*, 252–258.

[S8] K. M. Beeh, J. Beier, C. Lerch, A. K. Schulz, R. Buhl, *Lung* **2004**, *182*, 369–377.

[S9] M. A. Giembycz, *Expert Opin. Investig. Drugs* **2001**, *10*, 1361–1379.

[S10] R. Burch, T. Anderson, J. Lazar, *Capsaicinoid Gel Formulation and Uses Thereof.*, **2006**, US20060148903 A1.

[S11] H. Dinter, J. Tse, M. Halks-Miller, D. Asarnow, J. Onuffer, D. Faulds, B. Mitrovic, G. Kirsch, H. Laurent, P. Esperling, et al., *J. Neuroimmunol.* **2000**, *108*, 136–146.

[S12] S. Hanessian, S. Guesné, E. Chénard, *Org. Lett.* **2010**, *12*, 1816–1819.

[S13] M. J. Bonifácio, M. Archer, M. L. Rodrigues, P. M. Matias, D. a Learmonth, M. A. Carrondo, P. Soares-Da-Silva, *Mol. Pharmacol.* **2002**, *62*, 795–805.

[S14] E. Tsuji, K. Okazaki, M. Isaji, K. Takeda, *J. Struct. Biol.* **2009**, *165*, 133–139.

[S15] Y. J. Zheng, T. C. Bruice, *J. Am. Chem. Soc.* **1997**, *119*, 8137–8145.

[S16] D. Tsao, L. Diatchenko, N. V. Dokholyan, *PLoS One* **2011**, *6*, 1–5.

[S17] C. Zhang, R. L. Weller, J. S. Thorson, S. R. Rajski, *J. Am. Chem. Soc.* **2006**, *128*, 2760–2761.

[S18] G. Lukinavičius, V. Lapiene, Z. Staševskij, C. Dalhoff, E. Weinhold, S. Klimašauskas, *J. Am. Chem. Soc.* **2007**, *129*, 2758–2759.

[S19] W. Peters, S. Willnow, M. Duisken, H. Kleine, T. Macherey, K. E. Duncan, D. W. Litchfield, B. Lüscher, E. Weinhold, *Angew. Chemie - Int. Ed.* **2010**, *49*, 5170–5173.

[S20] R. Wang, W. Zheng, H. Yu, H. Deng, M. Luo, *J. Am. Chem. Soc.* **2011**, *133*, 7648–7651.

[S21] M. Thomsen, S. B. Vogensen, J. Buchardt, M. D. Burkart, R. P. Clausen, *Org. Biomol. Chem.* **2013**, *11*, 7606–7610.

[S22] S. Willnow, M. Martin, B. Lüscher, E. Weinhold, *ChemBioChem* **2012**, *13*, 1167–1173.

[S23] J. M. Winter, G. Chiou, I. R. Bothwell, W. Xu, N. K. Garg, M. Luo, Y. Tang, *Org. Lett.* **2013**, *15*, 3774–3777.

[S24] B. W. K. Lee, H. G. Sun, T. Zang, B. Ju-Kim, J. F. Alfaro, Z. S. Zhou, *J. Am. Chem. Soc.* **2010**, *132*, 3642–3643.
